# Deficiency of *Kif15* impairing synaptic development leads to mood disorder in mice

**DOI:** 10.1371/journal.pgen.1011839

**Published:** 2025-09-02

**Authors:** Xiaomei He, Wei Zhang, Xu Chen, Zhangji Dong, Chengyuan Wei, Tao Wu, Dexia Kong, Roujia Kong, Ronghua Wu, Yan Liu, Mei Liu

**Affiliations:** 1 Key Laboratory of Neuroregeneration of Jiangsu and Ministry of Education, Co-innovation Center of Neuroregeneration, Medical School, Nantong University, Nantong City, China; 2 Department of Basic Medicine, Nantong Health Vocational College, Nantong City, China; 3 Key Laboratory of Neuroregeneration of Jiangsu and Ministry of Education, Co-innovation Center of Neuroregeneration, Nantong University, Nantong City, China; 4 College of Xinglin, Laboratory Animal Center of Nantong University, Nantong City, China.; Cleveland Clinic Cole Eye Institute, UNITED STATES OF AMERICA

## Abstract

The harmony of neuronal excitation and inhibition is essential for precise neuronal circuitry in the developmental brain, and thus affects the human emotion. Abnormalities of synaptic morphology directly affect neuronal function and contribute to a variety of psychiatric disorders. Previous studies have shown that *Kif15* (Kinesin-12), a microtubule-associated motor protein, affects neurite growth, navigation, and branching during neuronal development, revealing the potential of *Kif15* to influence neuronal dendritic morphology. A GWAS study in a European population showed that there were variants in both exons and introns of the *KIF15* gene on chromosome 3 in patients with depression. Therefore, we generated *Kif15*^*-/-*^ mice using CRISPR/Cas9 technology. In this study, we found that *Kif15*^*-/-*^ mice have exhibited significant impacts on dendritic morphology and function, which contributes to mood disorders. Compared with *Kif15* wild-type mice, adolescent *Kif15*^*-/-*^ mice showed a significant decrease in the excitatory postsynaptic scaffolding protein PSD95 and NMDA receptors, as well as a reduction in the total density of dendritic spines and the density of mushroom spines, and a decrease in the frequency of mEPSCs. Meanwhile, the inhibitory postsynaptic scaffold protein Gephyrin and GABRB1 significantly upregulated. However, the adult *Kif15*^*-/-*^ mice simultaneously exhibited an obvious manic behavior and their PSD95 expression increased rapidly, even more than that of the *Kif15* wild-type mice. Meanwhile, overexpression of *Kif15* in *kif15*^*−/−*^ zebrafish rescued their depressive behavior. In terms of molecular mechanism, we showed that KIF15 interacted with PSD95 protein using both endogenous and exogenous Co-IP assays. Furthermore, we found that PSD95 in *Kif15*^*-/-*^ mice was distributed around neuronal nuclei, in contrast to PSD95 localized close to the cell membrane in *Kif15* wild-type mice. In conclusion, our study has identified a microtubule-associated molecular motor, KIF15, that plays a novel role in bipolar disorder through its contributions to spine morphology and function.

## Author summary

Mood disorders are a common mental illness with a high incidence rate. The etiology of mood disorders is complex, involving both genetic and environmental factors, although the precise cause remains unknown. Previous GWAS analysis showed KIF15 gene is a candidate gene for depression, but the molecular mechanism is unknown. In our study, we found that KIF15 gene knockout mice exhibited mood disorder during the postnatal development. Further investigation showed an imbalance in the expression of excitatory and inhibitory postsynaptic proteins at different stages, which may lead to bipolar disorder, characterized by alternating episodes of depression and mania. KIF15, also named kinesin-12, is a microtubule-associated motor protein, and our study revealed that KIF15 involved in the PSD95 transportation and localization at postsynaptic membrane, and thus the deficiency of KIF15 gene disrupt the localization of PSD95, and hence impair the expression of AMPA receptors and NMDA receptors in synapses. Our data uncover a novel function of KIF15 in synapses and also offer valuable insights into the pathogenesis of mood disorders.

## 1 Introduction

Mood disorders, which can be roughly divided into depression and bipolar disorder [1,2], often involve multiple brain regions, including the prefrontal cortex and hippocampus. The prefrontal cortex (PFC) is a highly intricate brain region both in terms of its structure and function, encompassing working memory, emotion regulation, sensorimotor, cognition and decision-making, as well as other advanced features [3–6]. Hippocampus is one of the neurogenesis areas of the adult brain, which is mainly involved in learning and memory, and also has certain cognitive and emotional regulation functions [7,8]. Dysfunction of the prefrontal cortex or hippocampus is often linked to a range of psychiatric conditions, such as schizophrenia, depression, and bipolar disorder [9,10]. In the prefrontal cortex, the postsynaptic membrane of most glutamatergic excitatory synapses is composed of spines on dendrites [11]. In contrast, inhibitory synapses mainly occur in the shaft of dendrites, neuron bodies, or axon initial segments [12]. The number and morphology of dendritic spines undergo changes throughout mouse development and adulthood, and this plasticity of dendritic spines is closely associated with synaptic function. Previous studies have indicated alterations in both the density and morphology of dendritic spines in the prefrontal cortex of a mouse model exhibiting depressive symptoms [13,14]. However, the specific molecular mechanism underlying the relationship between dendritic spines and the mood disorders is worthy of further investigation.

In the central nervous system, excitatory neurons modulate synaptic function by regulating postsynaptic scaffolding proteins such as PSD95 thereby altering neuronal excitability [15,16]. PSD95 is a member of the membrane-associated guanylate kinase (MAGUK) class of proteins in the synapse, which regulates the stabilization, recruitment and trafficking of postsynaptic glutamatergic receptors such as AMPA receptors and NMDA receptors to the postsynaptic membrane [17,18]. The postsynaptic scaffolding proteins at inhibitory synapses are mainly gephyrin, but the specific molecular mechanisms of how inhibitory postsynaptic scaffolding proteins and related receptor proteins are regulated have been less studied [19,20]. PSD95 deficiency causes an increase in the number of inhibitory GABAergic synapses and receptor expression, whereas overexpression of PSD95 causes a decrease in the number of inhibitory GABAergic synapses in the hippocampal neurons, thereby altering the ratio of excitatory to inhibitory synapses (E/I) [21,22]. A large number of studies have shown that both depression and bipolar disorder are associated with abnormal synaptic function [23,24]. Genome-wide association studies (GWASs) found that the variant risk genes of bipolar disorder were also mainly enriched in genes related to synaptic function [25].

Neurons are highly polarized cells, and intracellular material transport plays an important role in the maintenance of cell morphology and function. There are three main motor proteins involved in substance transport in neurons, including dynein, kinesin and myosin [26]. Kinesins transport a variety of proteins, mRNA or organelles along microtubules. A variety of kinesins have been reported to be involved in vesicular or postsynaptic protein trafficking [26,27]. *Kif15* is a member of kinesin12 family, which is an N-terminal plus-end-directed mitotic motor protein involved in the establishment of bipolar spindle, cell proliferation, apoptosis, and differentiation. It plays a crucial role in neuronal development [28,29]. Our previous study indicated that *Kif15* mediates axon growth, navigation, and branching through microtubule growth and sliding in neurons [28]. We previously reviewed a genome-wide association study (GWAS) [30] in major depressive patients of Europe, and identified the presence of SNP (Single nucleotide polymorphism) variants in the KIF15 gene on chromosome 3. The SNP variants of rs3804583, rs3804580 and rs7622843 are significantly above the threshold in control population. Therein, the rs3804583 was found to be located in the exon of the KIF15 gene, while rs3804580 and rs7622843 were found in the intron. (S1 Fig). Additionally, a SNP variant in the intron of KIF15 gene was also found in another GWAS [31] of depression patients from multi-ancestry. According to the NCBI, the SNP rs3804583, located in the exon, is mutated from C to A, G, or T at 44843155 (GRCh38) or 44884647 (GRCh37) on chromosome 3. These mutations occur at the same base site in the CDS region of the KIF15 gene. The mutation of this base led to a missense mutation of KIF15 protein, Leu1206Met or Leu1206Val. According to literature, the tail region of KIF15 consists mainly of amino acids 1149–1388 [32]. so this missense mutation primarily affects the tail function of KIF15. This region of kinesin is crucial for the binding of cargos [26,33]. Therefore, we propose that the mutation at this SNP ultimately disrupts the binding of KIF15 with its cargo. These findings suggest that the KIF15 gene may play a role in the onset or development of major depression. Therefore, we adopted the KIF15 knockout C57BL/6 mice to investigate whether *Kif15* gene involving in the emotional disorder and the possible molecular mechanism.

## 2 Result

### 2.1 *Kif15* deficiency impacts the development of dendritic spines in PFC and Hippocampus in mice

**Fig 1:**
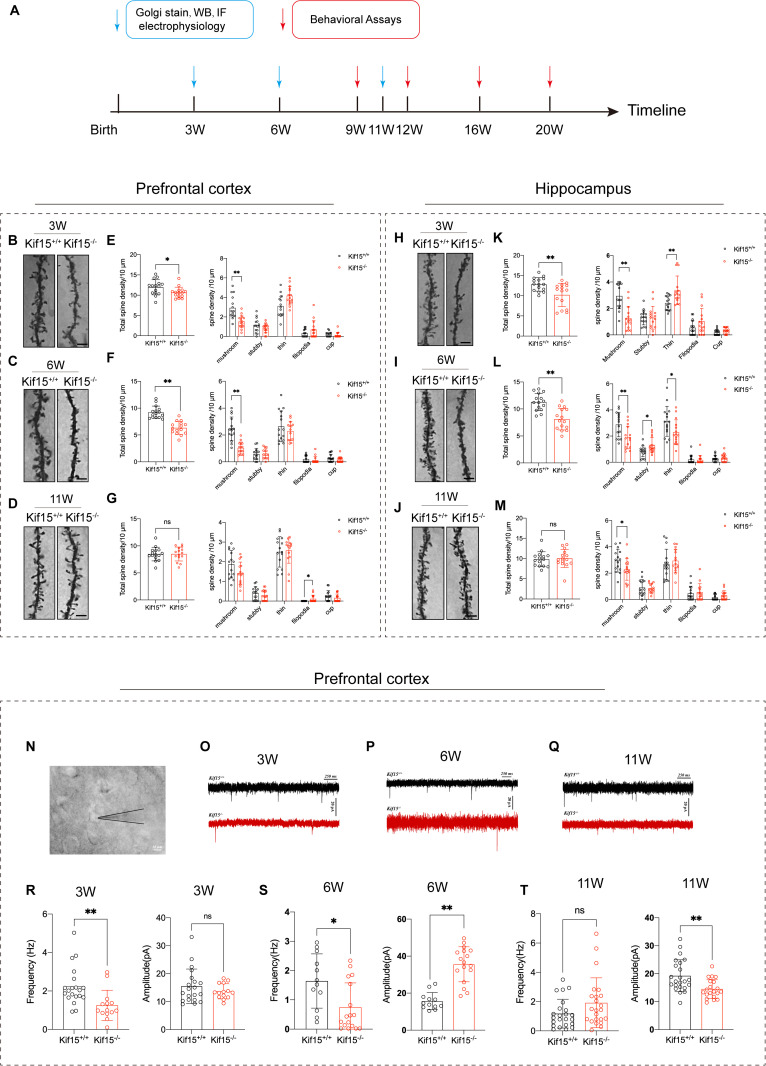
*Kif15* deficiency leads to impaired development of dendritic spines in PFC and hippocampus of mice. A: Experimental design. WB: Western Blot; IF: immunofluorescence staining. B-D: Representative images of neuronal dendrites by Golgi staining in layer2/3 neurons of PFC at 3,6 and 11 week-old *Kif15*^*+/+*^ and *Kif15*^*-/-*^ mice, Scale bar = 5μm. H-J: Representative images of neuronal dendrites by Golgi staining in CA1 of hippocampus at 3,6 and 11 week-old *Kif15*^*+/+*^ and *Kif15*^*-/-*^ mice, Scale bar = 5μm. B, C, E and F: Decreased densities of total spine and mushroom-like spines in the PFC neurons from *Kif15*^*-/-*^ in 3, 6 week-old mice, compared with *Kif15*^*+/+*^ mice, n = 15 dendrites of neurons from 3 mice in each group. H, I, K and L: Decreased densities of total spine and mushroom-like spines in the CA1 of hippocampus from *Kif15*^*-/-*^ in 3, 6 week-old mice, compared with *Kif15*^*+/+*^ mice, n = 15 dendrites of neurons from 3 mice in each group. D and G: Similar total dendritic spine density between *Kif15*^*+/+*^ and *Kif15*^*-/-*^ in 11 week-old mice expect for filopodia in PFC. J and M: Similar total dendritic spine density between *Kif15*^*+/+*^ and *Kif15*^*-/-*^ in 11 week-old mice expect for mushroom like spine in hippocampus. The densities of different types of dendritic spines in PFC neurons from different weekly mice were quantified, n = 15 dendrites of neurons from 3 mice in each group. N: Diagram to show the whole-cell recording of pyramidal neurons in layer 2/3 of PFC from *Kif15*^*+/+*^ and *Kif15*^*-/-*^ mice. **(O-Q)**: Representative mEPSCs traces of neurons in PFC from 3, 6 and 11 week-old mice. R: Decreased mEPSCs frequency and similar amplitude of neurons in 3 week-old, n = 20 neurons from 6 *Kif15*^*+/+*^ mice, n = 14 neurons from 5 *Kif15*^*-/-*^ mice. S: Decreased frequency but increased amplitude of mEPSCs in PFC neurons in 6 week-old *Kif15*^*-/-*^ mice, compared with *Kif15*^*+/+*^ mice, n = 12 neurons from 5 *Kif15*^*+/+*^ mice, n = 17 neurons from 5 *Kif15*^*-/-*^ mice. T: Similar frequency and decreased amplitude of mEPSCs in PFC *Kif15*^*-/-*^ mice at 11 week old, n = 23 neurons from 4 *Kif15*^*+/+*^ mice, n = 23 neurons from 4 *Kif15*^*-/-*^ mice. All the data are presented as mean±SD, *P < 0.05, **P < 0.01,two-tailed student’s t test between two groups, Multiple t test performed in different morphology spine group.

Our previous study reported that *Kif15* knockout mice did be prone to depression [34], and the basic morphology and molecular mechanism need to further clarify. Mice of depressive models typically exhibit reduced dendritic spine density [35]. Thus, we detected the dendritic spine density by Golgi staining, and the data showed that *Kif15* knockout mice exhibited the developmental delay pf dendritic spine from postnatal 3 w to 11 w. Specifically, *Kif15*^*-/-*^ mice show reduced total dendritic spine density and a lower density of mature mushroom-like spines compared to wild-type mice at postnatal 3 weeks (Fig 1B, 1C, 1E, 1F) and 6 weeks (Fig 1H, 1I, 1K, 1L) of development in PFC and hippocampus. The findings suggest that *Kif15* plays a significant role in the development and maturation of dendritic spines during adolescence. By 11 weeks, although total dendritic spine and mushroom-like spine densities in *Kif15*^*-/-*^ mice had normalized to wild-type levels, the filopodia spine densities were still higher in PFC (Fig 1D, 1G). In the hippocampus, although the total dendritic spines were similar to that of the wild type, the density of mushroom dendritic spines in *Kif15*^*-/-*^ mice of 11 weeks old was still less than that in WT mice(Fig 1J,1M), suggesting that the *Kif15*^*-/-*^ mice still had developmental delay in adulthood.

**Fig 2:**
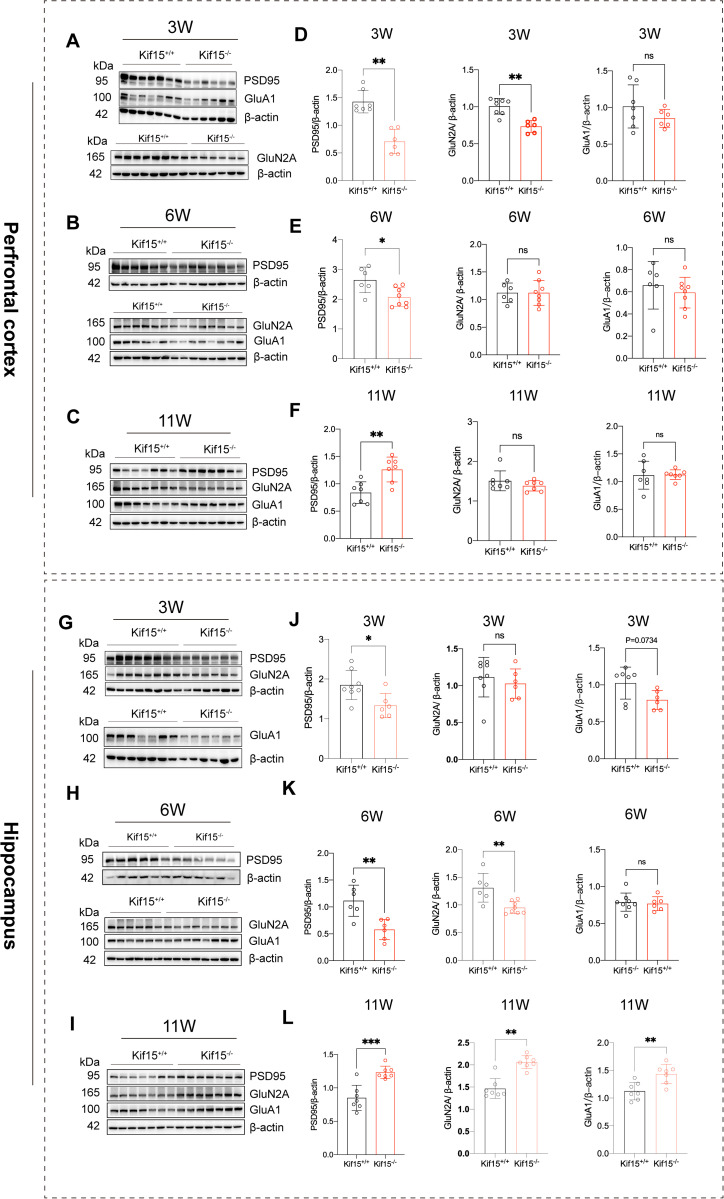
*Kif15* deficiency affects the expression of excitatory postsynaptic receptors in PFC and hippocampus. **(A and D)**: Western Blot analysis showed decreased protein levels of PSD95 and NMDA receptor GluN2A in the PFC of 3 week-old *Kif15*^*-/-*^ mice, n = 7 ~ 8 in *Kif15*^*+/+*^ group, n = 6 in *Kif15*^*-/-*^ group. **(B and E)**: Decreased expression of PSD95 in the PFC at 6 week-old *Kif15*^*-/-*^ group mice, n = 6 in *Kif15*^*+/+*^ group, n = 8 in *Kif15*^*-/-*^ group. **(C and F)**: Increased expression of PSD95 in the PFC of *Kif15*^*-/-*^ group compared with *Kif15*^*+/+*^ group mice at 11 week-old, n = 7 in each group. **(G and J)**: The expression of PSD95 in hippocampus of *Kif15*^*-/-*^ mice decreased at 3 weeks, n = 8 in *Kif15*^*+/+*^group, n = 6 in *Kif15*^*-/-*^ group (**H and K**): The expression of PSD95 and GluN2A in hippocampus of *Kif15*^*-/-*^ mice was lower than that of wild type mice at 6 weeks, n = 6 in *Kif15*^*+/+*^ group, n = 8 in *Kif15*^*-/-*^ group. **(I and L)**: The expression of PSD95, GluA1 and GluN2A in the hippocampus of *Kif15*^*-/-*^ mice was significantly higher than that of *Kif15* WT mice at 11 weeks, n = 7 in each group. All the data are presented as mean±SD, *P < 0.05, **P < 0.01, two-tailed student’s t test were used for comparison between two groups, expect for the GluA1 of J, which was used as a nonparametric test.

**Fig 3:**
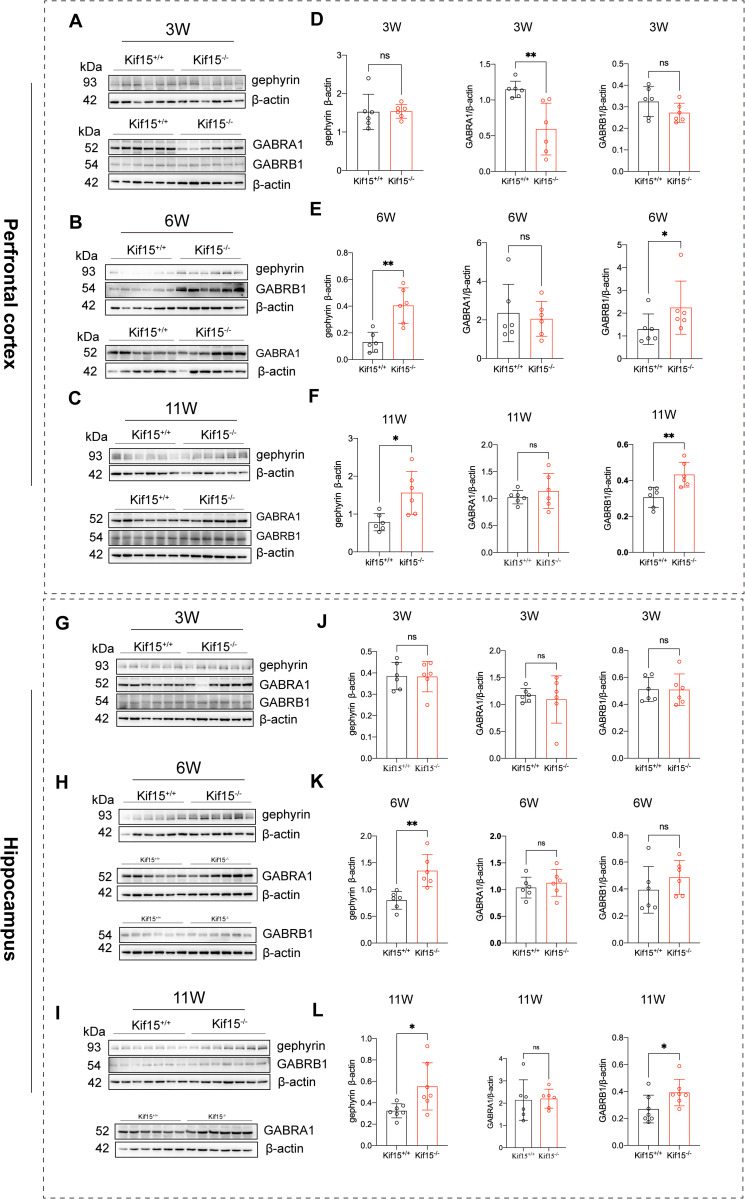
*Kif15* deficiency affects the expression of Gephyrin and GABRB1 in PFC and hippocampus. A-C: Western blot analysis of inhibitory postsynaptic proteins expression in 3, 6 and 11 week-old *Kif15*^*+/+*^ and *Kif15*^*-/-*^ mice in PFC. G-I: Western blot analysis of inhibitory postsynaptic proteins expression in 3, 6 and 11 week-old *Kif15*^*+/+*^ and *Kif15*^*-/-*^ mice in Hippocampus. D and J: Similar expression of gephyrin between two groups at 3 week-old in both PFC and hippocampus, n = 6 mice in each group. E and K: Increased gephyrin or GABRB1 expression in *Kif15*^*-/-*^ mice compared with *Kif15*^*+/+*^ mice at 6 week-old in PFC and hippocampus, n = 6 mice in each group. F and L: High expression of gephyrin and GABRB1 in *Kif15*^*-/-*^ mice compared with *Kif15*^*+/+*^ mice in PFC and hippocampus, n = 6 mice in each group. All the data are presented as mean±SD, *P < 0.05, **P < 0.01, two-tailed student’s t test were used for comparison between two groups, expect for the GABRB1 of E, which was used as a nonparametric test.

**Fig 4:**
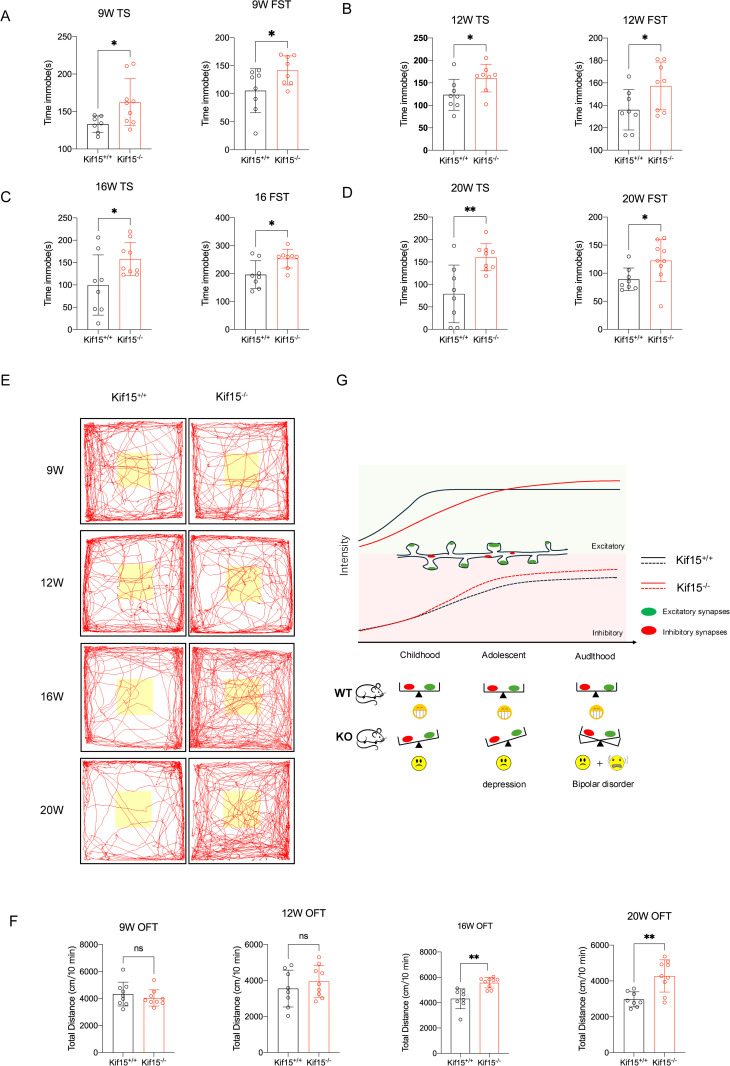
*Kif15* deficient mice exhibit both depressive and manic behaviors in adulthood. A-D: *Kif15*^*-/-*^ mice exhibited longer immobility times in Tail suspension test and the forced swimming test at 9, 12, 16, and 20 week-old, n = 7 or 8 in WT group, n = 8 or 9 in *Kif15*^*-/-*^ group; E: Representative tracking path for *Kif15*^*-/-*^ and *Kif15* WT mice during 10 minutes open field test in 9, 12, 16 and 20 week-old. F: *Kif15*^*-/-*^ mice traveled further in the 10 minutes open field test at 16 and 20 week-old; n = 8 in WT group, n = 9 in *Kif15*^*-/-*^ group, G: Schematic of the mechanism by which *Kif15*^*-/-*^ mice exhibit depression and mania. The image was created with Microsoft Power Point, and some icons are free downloaded from OPENCLIPART (https://openclipart.org/). All the data are presented as mean±SD, *P < 0.05, **P < 0.01,two-tailed student’s t test between two groups.

**Fig 5:**
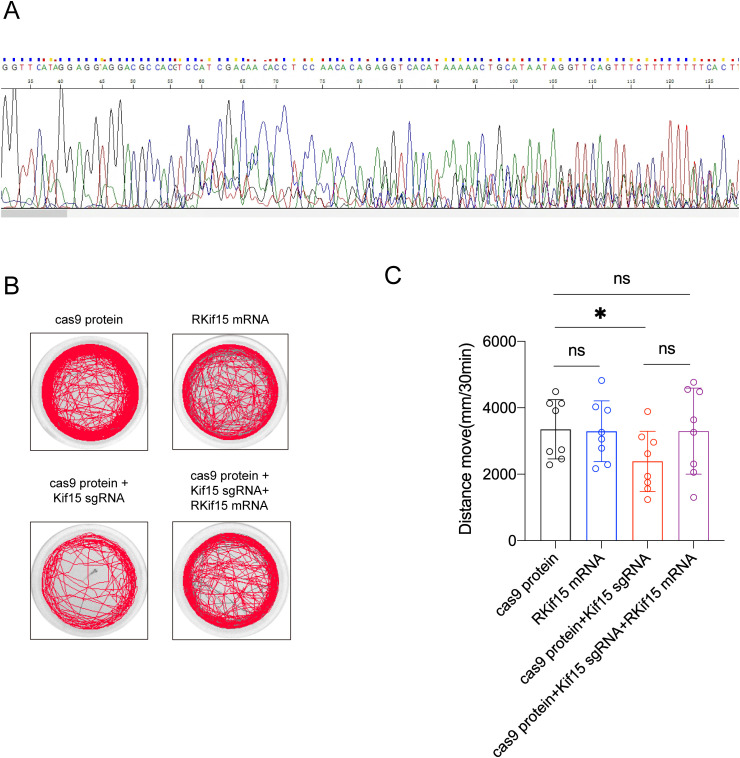
The depressive behavior in *Kif15*^*-/-*^ zebrafish was rescued following *Kif15* supplementation. A: Sequencing results of *Kif15*^*-/-*^ zebrafish. B: Representative plots of spontaneous swimming trajectories of zebrafish from different groups on day 4 after fertilization. C: On day 4 after fertilization, *Kif15* overexpression rescued the depressive behavior induced by *kif15* knockout, with 8 zebrafish larvae in each group. All the data are presented as mean±SD, *P < 0.05, **P < 0.01,two-tailed student’s t test between two groups.

The frequency and amplitude of miniature excitatory postsynaptic currents (mEPSCs) are used to evaluate the synaptic activity [36]. We thus performed the Whole-cell patch clamp recordings of neurons (Fig 1N) in PFC to detect the synaptic function and the results were shown in Fig 1O-1Q. As the statistical data shown in Fig 1R and 1S, the mEPSCs frequency of *Kif15*^*-/-*^ mice showed significant decreases at postnatal 3 w and 6 w, which were consistent with the number reduction of the total dendritic spines.

Surprisingly, while there was no difference in mEPSCs amplitude at 3 weeks (Fig 1O and 1R), the amplitude in the *Kif15*^*-/-*^ group was significantly higher than in the wild-type group at 6 weeks (Fig 1P and 1S). This suggests that the function of postsynaptic receptors may be affected during adolescence. Additionally, at 11 weeks, mEPSCs amplitudes in the PFC of *Kif15*^*-/-*^ mice were significantly lower than those in *Kif15*^*+/+*^ mice, and the frequency was not different from *Kif15*^*+/+*^ mice (Fig 1Q and 1T). The amplitude of mEPSCs is related to the expression and properties of excitatory postsynaptic receptors [37]. Therefore, we detected the receptors’ expression accordingly.

### 2.2 *Kif15* deficiency impacts the expression patterns of PSD95 and glutamate receptors in the PFC and hippocampus in mice

These above results revealed that *Kif15* contributes to the development and function of dendritic spines. We then investigate whether *Kif15* gene deficiency affects the expression of PSD95 and NMDA or AMPA receptors. The postsynaptic density protein 95 (PSD95) involves in maturation of excitatory synapses, which are related to emotion [38]. Previous studies have reported that NMDA and AMPA receptors are altered in depressive rodents or patients [39,40], therefore, we detected the changes of GluN2A and GluA1 proteins. The results showed that the PSD95 protein in the prefrontal cortex and hippocampus of *Kif15*^*-/-*^ mice significantly reduced at postnatal 3 w and 6 w(Fig 2A, 2B, 2D, 2E and 2G, 2H, 2J, 2K). Meantime, the GluN2A were decreased at 3 or 6 w(Fig 2A, 2D and 2H, 2K). Specifically, at postnatal 3 w, GluN2A expression reduced in the prefrontal cortex (Fig 2A and 2D), while the expression of GluA1 was also tend to decreased in the hippocampus (Fig 2G and 2J). At 6 w, there was no significant difference in the expression of GluN2A in PFC between the *Kif15* WT and KO mice (Fig 2B and 2E), but GluN2A still decreased in the hippocampus(Fig 2H and 2K). These data demonstrated that *Kif15* exerted a more pronounced influence on the GluN2A, and its expression alterations exhibited distinct temporal dynamics across various brain regions, potentially associated with the diverse regional expression patterns of GluN2A. Surprisingly, at 11 weeks of adulthood, the expression of PSD95 in the hippocampus and prefrontal cortex of *Kif15* KO mice was higher than that of *Kif15*^*+/+*^ mice(Fig 2C, 2F and 2I, 2L), and the expression of GluN2A and GluA1 in the hippocampus was increased simultaneously (Fig 2I and 2L). This phenomenon may be attributed to crosstalk of different brain areas following the developmental process.

### 2.3 *Kif15* deficiency impacts the expression patterns of the inhibitory postsynaptic scaffolding protein Gephyrin and receptor protein GABRB1

The imbalance of excitation and inhibition (E/I) in the prefrontal cortex often results in the mood disorder [41]. Our preliminary data suggest that *Kif15*^*-/-*^ mice begin exhibiting depressive tendencies at 6 weeks-old. We hypothesized that, in addition to reduced expression of excitatory synapse-associated proteins, inhibitory postsynaptic proteins might also be affected. As we known, GABA acts primarily through activation of two different receptors, including ionic GABA_A_ receptors and metabolic GABA_B_ receptors, whereas its signaling function is mainly mediated by type A ionic receptors, so we chose two subunits of GABA_A_Rs, GABA_A_Rα1 and GABA_A_Rβ1 receptor subunits [42]. Western-blotting analysis revealed no significant difference in gephyrin expression between *Kif15*^*-/-*^ and *Kif15*^*+/+*^ mice at 3 weeks (Fig 3A, 3D and 3G, 3J), However, gephyrin expression both in the prefrontal cortex and hippocampus of *Kif15*^*-/-*^ mice was significantly elevated compared to controls at 6 weeks (Fig 3B, 3E and 3H, 3K), and this increased expression persisted through to 11 weeks of adulthood (Fig 3C, 3F and 3I, 3L). At the same time, the expression of the inhibitory postsynaptic receptor γ-aminobutyric acid type A receptor β1 was increased in the prefrontal cortex and hippocampus of adult *Kif15*^*-/-*^ mice in adulthood (Fig 3C, 3F and 3I, 3L). However, the upregulation of GABRB1 expression was observed only in the prefrontal cortex at 6 weeks (Fig 3B and 3E), suggesting that *Kif15* deficiency has region-specific effects on GABRB1 expression changes at different time points. Meantime, the expression of GABRA1 in the PFC of *Kif15*^*-/-*^ mice was significantly lower than that of *Kif15*^*+/+*^ mice at 3 weeks (Fig 3A and 3D), and there was no difference between the two groups in either the PFC or the hippocampus at later stages of development(Fig 3B, 3C and 3E–3L), suggesting that *Kif15* only influences GABRA1 expression in the PFC during the childhood. Previous studies have reported that GABRB1 is associated with bipolar disorder [42], and our data also revealed the notable increases of the inhibitory postsynaptic scaffolding protein Gephyrin and inhibitory receptor of GABRB1 in the *Kif15*^*-/-*^ mice.

### 2.4 Adult *Kif15*^*-/-*^mice exhibit both depressive and manic behaviors

The above results showed both the expressions of excitatory and inhibitory postsynaptic receptors increased in adult *Kif15*^*-/-*^ mice. Previous studies reported that patients with bipolar disorder have increased neuronal excitability [43] and abnormal expression of γ-aminobutyric acid or its receptor [44]. Accordingly, we speculated whether such bidirectional fluctuations in mood would also be present in *Kif15*^*-/-*^ mice. Since the expression of both excitatory and inhibitory proteins is increased in *Kif15* KO mice at 11 weeks, we simultaneously examined the depressive and manic behaviour of the mice before and after 11 weeks (Fig 4). Combined with previous results from our group, we found that *Kif15*^*-/-*^ mice are depressed from 9W to 20 weeks. The immobility time of *Kif15*^*-/-*^ mice in the forced swim test and tail suspension test was significantly longer than that of *Kif15*^*+/+*^ mice, suggesting that the depressive behaviour of *Kif15*^*-/-*^ mice persists from adolescence to adulthood (Fig 4A-4D). In the open field test, we found that *Kif15*^*-/-*^ mice did not show manic behaviour until 16 weeks (Fig 4E and 4F), and the total distance moved was significantly higher than that of *Kif15*^*+/+*^ mice, whereas *Kif15* KO mice did not show manic behaviour around 11 weeks, including 9 weeks and 12 weeks(Fig 4E and 4F). This suggests that there may be some delay in the behavioural manifestation of molecular expression levels. In addition, the total distance traveled in the open field by *Kif15*^*-/-*^ mice was comparable to that of *Kif15*^*+/+*^ mice at 24 weeks (S4B Fig), and manic behavior was reduced, suggesting that manic-like behavior in *Kif15*^*-/-*^ mice may primarily emerge in early adulthood. Furthermore, the time spent in and frequency of entries into the open arm of the elevated maze were significantly lower in *Kif15*^*-/-*^ mice compared to *Kif15*^*+/+*^ mice at 14 weeks of age. These mice exhibited anxiety-like behavior, which persisted until 9 months (S4A Fig), indicating that the emotional changes caused by *Kif15* deletion in mice become more complex and varied with age. Our data also suggested that the reduced expressions of excitatory postsynaptic receptors in younger *Kif15*^*-/-*^ mice, and their rapidly increased in adult *Kif15*^*-/-*^ mice.

### 2.5 The depressive behavior in *Kif15*^*-/-*^ zebrafish was rescued by *Kif15* gene supplementation

We used CRISPR/Cas9 to generate *kif15* full knockout zebrafish and established a rescue group that overexpressed *Kif15* mRNA simultaneously with the knockout(Fig 5). Sequencing analysis of zebrafish co-injected with cas9 protein and sgRNA kif15 revealed multiple peaks downstream of the sgRNA site, indicating successful targeting and confirming the *Kif15*^*-/-*^ mutation(Fig 5A). Subsequent behavioral results showed that, at 4 days post-fertilization, the *kif15* knockout zebrafish exhibited significantly reduced movement compared to the control group, displaying depressive-like behavior. This depressive behavior was alleviated in the rescued group, where *Kif15* was overexpressed. In the overexpression of *Kif15* alone group, the distance traveled by the zebrafish was comparable to that of the control group(Fig 5B and 5C). These results further support the critical role of *kif15* deletion in the manifestation of depressive behavior.

### 2.6 KIF15 interacts with PSD95 and influences its intracellular localization

**Fig 6:**
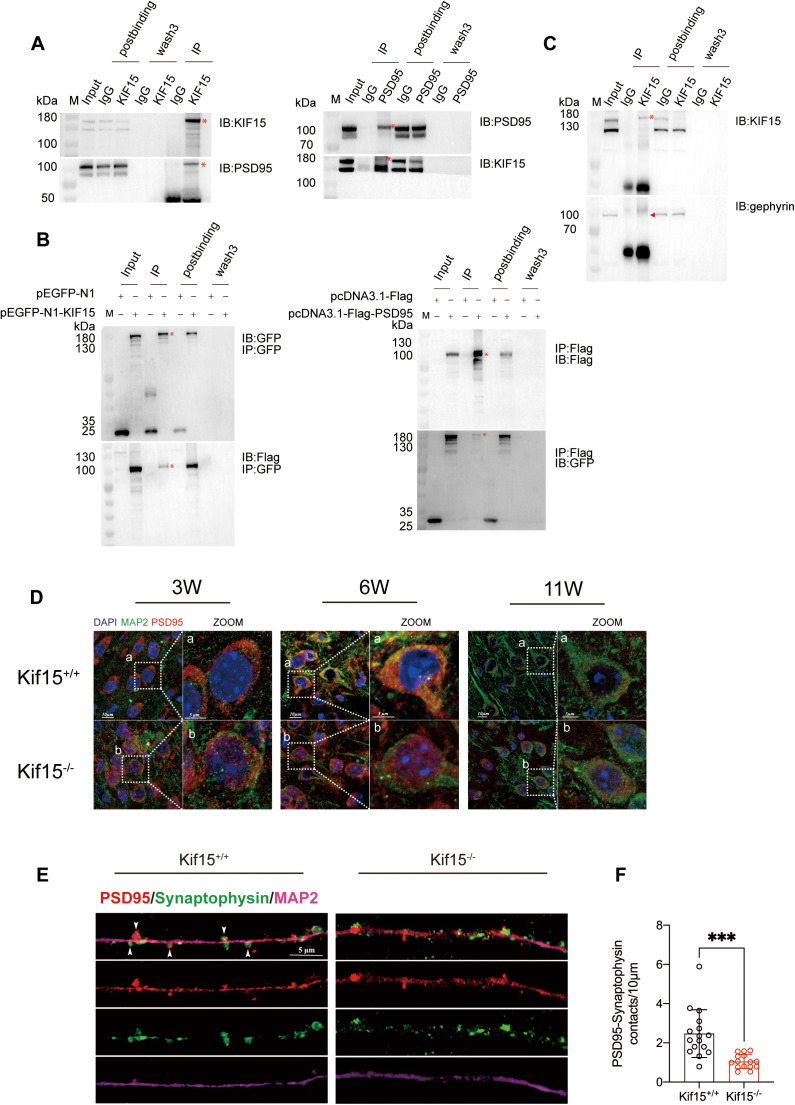
KIF15 interacts with PSD95 and affect its localization in neuronal cytoplasm neurons of the PFC. A and C: Co-IP result of cortical protein homogenate from P1 *Kif15*^*+/+*^ mouse. B: Result of co-IP assays from 293T cells which simultaneous overexpression of KIF15-GFP and PSD95-Flag. Asterisks indicate interaction bands in the Western blot assays, triangles represent negative bands with no interaction. D: Immunofluorescent images showing the distribution of PSD95 expression in the PFC neurons at 3, 6 and 11 week-old mouse (Right panel, higher magnification of dashed boxed area). E: Wildtype and Kif15 knockout DIV18 neurons were immunostained for PSD95 and presynaptic synaptophysin. The colocalization of PSD95 and synaptophysin in confocal images was considered as a synapse (solid white arrows). F: The synaptic density in KIF15 knockout neurons was significantly lower compared to the wildtype group, n = 15 neurons per condition. All the data are presented as mean±SD, ***P < 0.001,two-tailed student’s t test between two groups.

**Fig 7:**
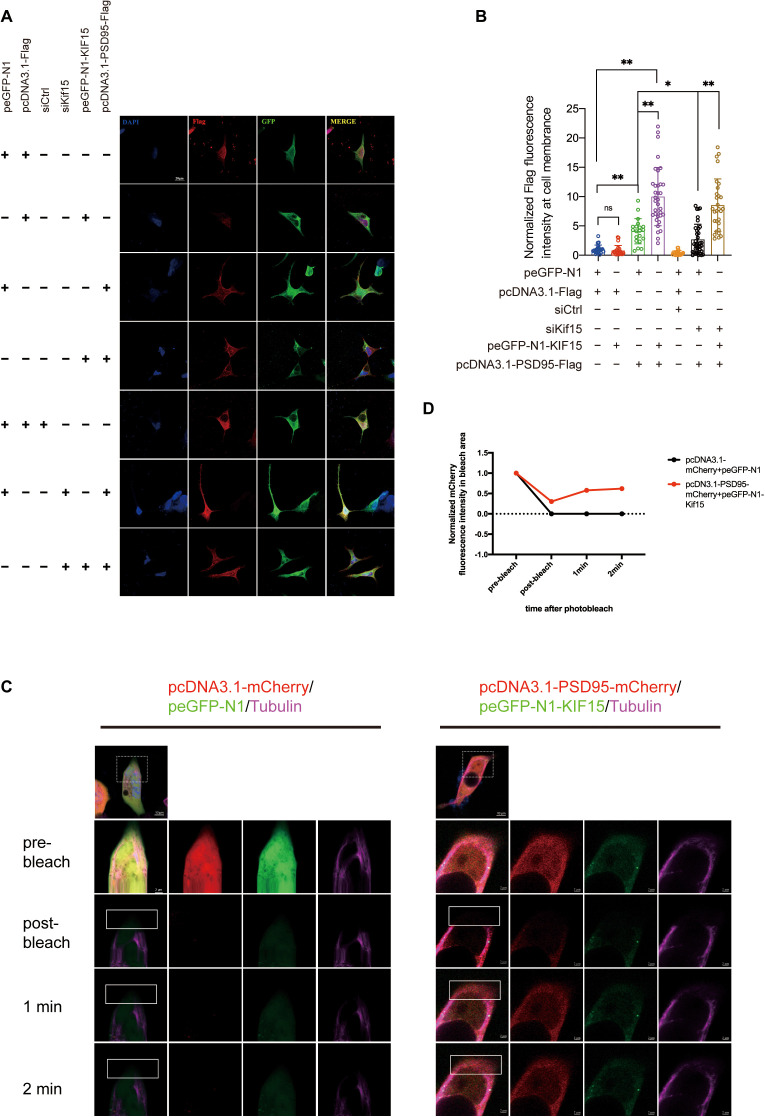
KIF15 mediates the trafficking of PSD95 along microtubules. A and B: Overexpression or knockdown of *Kif15* in 293T cells affected the expression and distribution of PSD95 on the cell membrane. n ≥ 20 neurons in each group, All the data are presented as mean±SD, *P < 0.05, **P < 0.01,two-tailed student’s t test between two groups. C: FRAP analysis of fluorescence recovery of PSD95-mCherry in 293T cells, The white dashed box in the upper panel is the magnified area of the image in the lower panel, and the white solid box is the bleached area. D: Fluorescence recovery intensity in the bleached region (white solid-line box) was measured at fixed time intervals.

**Fig 8:**
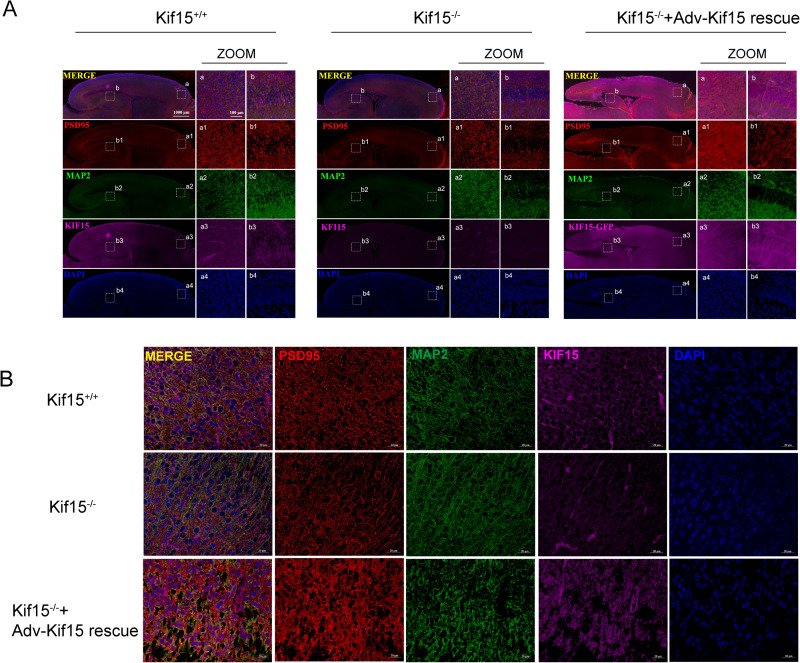
Intrauterine supplementation of *Kif15* in E15 *Kif15*^*-/-*^ mice rescued the distribution of PSD95 in *Kif15*^*-/-*^ PFC neurons. A: Changes in PSD95 expression in the cortex and hippocampus of P1 mice were observed following cortical injection of adenovirus overexpressing *Kif15* into the cortex of E15 *Kif15*^*-/-*^ embryos. The right panel is a magnified view of the white dashed box in the left panel. B: The abnormal distribution of PSD95 within neurons in the PFC region of P1-day *Kif15*^*-/-*^mice was rescued by cortical injection of adenovirus overexpressing *Kif15* into E15 *Kif15*^*-/-*^ embryos. Bar = 20 μm.

We next investigated the relationship of KIF15 and PSD95. As a microtubule dependent motor protein, KIF15 involves in various cellular process, including spindle organization, axonal growth and intracellular transport. We suggested KIF15 may interact with PSD95, and facilitates its intracellular transportation and localization at postsynaptic membrane. The Co-immunoprecipitation (Co-IP) result revealed an interaction between endogenous KIF15 and PSD95 (Fig 6A). To further confirm the interaction, we co-transfected GFP-KIF15 and PSD95-Flag plasmids into 293T cells and performed the exogenous Co-IP. The results demonstrated that KIF15 and PSD95 interacted with each other (Fig 6B). Since the GWAS study suggested that the rs3804583 mutation may affect the binding ability of KIF15 to its cargo, we hypothesized that this mutation would impact the binding efficiency of KIF15 to PSD95. To test this, we introduced point mutations (C3636A and C3636G) at the corresponding sites in the wildtype Kif15 plasmid and evaluated its binding efficiency with PSD95 in 293T cells. The results indicated that both the C3636A and C3636G mutants exhibited significantly reduced interaction with PSD95-Flag compared to the wildtype control group, suggesting that mutations at this site may impact the binding ability between KIF15 and PSD95 (S1B and S1C Fig). The kinesin superfamily, including KIF15, is crucial for the intracellular transport of various substances such as protein complexes, mRNAs, and membrane organelles [45]. To determine if KIF15 influences PSD95 localization in neurons, we performed immunofluorescence test. The results revealed that in the prefrontal cortex of *Kif15*^*-/-*^ mice, PSD95 in the neuronal cytoplasm was predominantly clustered around the nucleus compared to *Kif15*^*+/+*^ mice, with the most pronounced difference observed at 6 weeks (Fig 6D). To further examine the postsynaptic distribution of PSD95, we performed immunofluorescence staining for PSD95 and presynaptic synaptophysin in wildtype and Kif15 knockout neurons at DIV18. The results showed that neurons from KIF15 knockout mice exhibited a remarkable reduction in synaptic density compared to wildtype neurons, suggesting that the distribution of PSD95 in the postsynaptic membrane was also significantly reduced (Fig 6E and 6F). Meanwhile, we treated cultured wildtype neurons with a KIF15 inhibitor to examine the distribution of PSD95, and obtained the similar results (S5A and S5B Fig). In addition, quantitative analysis of PSD95 fluorescence in the dendrites of neurons in the PFC region revealed that PSD95 fluorescent particles were retained in the dendrites of neurons in *Kif15*^*-/-*^ mice at three time points, compared to *Kif15*^*+/+*^ mice(S5C and S5D Fig). This suggests that the absence of KIF15 significantly disrupts PSD95 localization in neurons. This suggests that *Kif15* may play a role in transporting PSD95 along microtubules. In addition, we examined the intracellular localization of PSD95 following the overexpression or knockdown of *Kif15* in 293T cells. The results revealed that, upon overexpression of *Kif15*, PSD95 was significantly enriched at the cell membrane. Conversely, knockdown of *Kif15* notably reduced membrane-localized PSD95, a reduction that could be rescued by overexpressing *Kif15*(Fig 7A and 7B). Live cell imaging FRAP experiments showed that when both KIF15 and PSD95 were overexpressed, the fluorescence recovery on microtubules after bleaching was significantly stronger compared to the control group (Fig 7C and 7D). Subsequently, *Kif15*^*-/-*^ mice were injected with adenovirus overexpressing *Kif15* into the embryonic cortex of E15 *Kif15*^*-/-*^ mice, and the expression of PSD95 in the prefrontal cortex (PFC) was assessed by immunofluorescence on P1(Fig 8). Compared to *Kif15*^*-/-*^ mice, the rescue group exhibited a more uniform distribution of PSD95 in neurons (Fig 8B). Additionally, we observed that PSD95 expression was higher in the rescue group than in both the *Kif15*^*+/+*^ and *Kif15*^*-/-*^ groups in the PFC and hippocampus (Fig 8A). These findings suggest that *Kif15* not only influences the localization of PSD95 but also plays a role in regulating its expression. In addition, Co-IP analysis of P1 wild mice PFC showed that KIF15 did not interact with gephyrin (Fig 6C). It has been reported that depletion of PSD95 can increase the number of GABAergic synapses and the expression of GABAergic receptors. Therefore, we hypothesized that the increased expression of inhibitory postsynaptic proteins caused by the decreased expression of PSD95 resulted from *Kif15* deficiency.

## 3 Discussion

In this study, the effects of *Kif15* deficiency on the development of dendritic spines in the prefrontal cortex and hippocampus of mice were revealed. Meanwhile, the loss of *Kif15* leads to an imbalance in the expression of excitatory and inhibitory synapses throughout the development of mice. Due to the deficiency of *Kif15*, the expression of excitatory postsynaptic scaffold protein PSD95 is decreased, while the expression of inhibitory postsynaptic scaffold protein gephyrin is increased during adolescence, and *Kif15*^*-/-*^ mice mainly exhibit depressive behavior. After adulthood, the expressions of excitatory and inhibitory synaptic proteins in *Kif15*^*-/-*^ mice increased, which may be one of the reasons why *Kif15*^*-/-*^ mice develop bipolar disorders like behavior. In addition, overexpression of *Kif15* in *Kif15*^*-/-*^ zebrafish reversed the depression-like behavior, further confirming the important role of kif15 in depression-like behavior. It is evident that *Kif15*, as a microtubule plus-end-directed protein, plays an indispensable role in the developmental process for the transport on dendrites or the anchoring of postsynaptic membranes.

Therefore, we propose that KIF15 plays a crucial role in the development of excitatory synapses in the prefrontal cortex of mice and influences the balance between excitatory and inhibitory synaptic expression. As a member of the kinesin superfamily, *Kif15* is essential for neuronal development. It affects PSD95 localization in the neuronal cytoplasm through interaction with PSD95, which may contribute to the impaired dendritic spine density and morphology observed in *Kif15*^*-/-*^ mice. In addition, NMDA receptors can directly bind to PSD95, but AMPA receptors do not [46,47], so the reduction of PSD95 caused by *Kif15* deficiency may directly affect NMDA receptor expression or membrane anchoring.

In addition, at 6 weeks, the whole-cell patch clamp results showed that the frequency of *Kif15*^*-/-*^ mice decreased and the amplitude of mEPSCs increased. The amplitude of mEPSCs represents the change of the number or nature of glutamatergic receptors, but the protein expression of two excitatory postsynaptic receptors in the prefrontal cortex of *Kif15*^*-/-*^ mice was not different from that of the wild group. Here it is more likely to affect the synaptic function. Surprisingly, at 11 weeks, there was no difference in the frequency of mEPSCs between the two groups, but the amplitude of mEPSCs was significantly lower than that of WT mice, reflecting the complexity of organism development and the importance of *Kif15* in the development of mouse nervous system.

It has been reported in the literature that reduced ratios of excitability and inhibition are exhibited in rodents with major depression or depressive behavior [41,48]. Our previous results showed that *Kif15*^*-/-*^ mice already exhibit depressive tendencies during adolescence. In the present study, we examined both the excitatory postsynaptic scaffolding protein PSD95 and the inhibitory scaffolding protein gephyrin, and found that PSD95 was decreased in the *Kif15*^*-/-*^ mice during adolescence, whereas the expression of gephyrin was increased, and we speculate that this imbalance between excitatory and inhibitory synaptic expression is one of the reasons for triggering depressive-like behavior in *Kif15*^*-/-*^ mice, Moreover, the high expression of this inhibitory postsynaptic protein persisted into adulthood.

Therefore, adult *Kif15*^*-/-*^ mice not only change the expression of excitatory postsynaptic receptors, but also alter inhibitory postsynaptic receptors. It has been reported that patients with bipolar disorder have increased neuronal excitability and abnormal expression of γ-aminobutyric acid or its receptor. Therefore, we wondered whether *Kif15*^*-/-*^ mice also have such emotional fluctuations, and the results of behavioral experiments actually supported our suggestion. Behaviorally, the depressive behavior of *Kif15*^*-/-*^ mice also persisted from adolescence to adulthood. Although we detected increased expression of both excitatory and inhibitory proteins at 11 weeks, they did not show manic behavior until 16 weeks, and the mood swings of this depressive and manic behavior continued until 20 weeks. This not only reflects the consistency between molecular expression and behavior, but also suggests that the behavior is delayed. It has been reported in the literature [49] that patients with major depression often develop bipolar disorder with intermittent episodes of manic behavior, which may coincide with this biological mechanism we have revealed. Meanwhile, we will continue to study the mood swings of the mice after 20 weeks and even into old age.

In a follow-up study, we further explored how *Kif15* deficiency impacts dendritic spine morphology and subsequent emotional changes. Our preliminary results indicate that KIF15 interacts with the fourth class of cytoskeletal proteins, Septin7 and Septin3, the former are involved in maintaining cell morphology specifically [50]. Previous studies have shown that Septin7 interacts with PSD95 to influence dendritic spine morphology and synaptic distribution [51]. Additionally, Septin3 is a neuron-specific protein, suggesting that *Kif15* may play a role in mediating synaptic development through interactions with the Septins family proteins.

As a plus-end microtubule kinesin in neurons, *Kif15* not only affects the growth of neuronal axons, but also mediates the expression and localization of postsynaptic protein in vivo, and then affects the mood disorders in mice. In addition, other kinesins are involved in PSD95 trafficking in neurons, including KIF5A, a member of the Kinesin-1 family, which mediates the transport of PSD95 on dendrites by binding to the C-terminal region of its tail. [27]. It has also been reported that KIF5B plays a crucial role in the localization of PSD95 on dendrites and in dendritic spine plasticity [52]. Beyond that, our previous studies have found that the main difference between KIF15 and KIF11 is that KIF15 can interact with actin and plays a role in maintaining the growth cone and branching of protrusions. However, the unique role of KIF15, distinct from KIF5A and KIF5B in transporting PSD95, was not thoroughly explored in this study, which represents a limitation of the research. In summary, our study revealed a novel role of *Kif15* on the emotion, and also provided a new clinical target for the pathogenesis of bipolar disorder.

## 4 Materials and methods

### 4.1 Ethics statement

All mice experiments were conducted in accordance with the requirements of National Institutes of Health’s Guide for the Care and Use of Laboratory Animals and were approved by Nantong University. The studies were also reviewed and approved by institutional animal care and use committee(IACUC) of the Animal Center of Nantong University with approval number S20230408-012.

### 4.2 Details of Materials and methods are in supplementary information

#### 4.2.1 Animals and genotype analysis.

*Kif15*^*-/-*^ mice were *Kif15* systemic knockout mice made by CRISPR/Cas9 technology using C57BL/6 as background mice. In our breeding colony, two heterozygous females and one heterozygous male were mated to obtain homozygotes. All animals were housed in a constant environment where was maintain on a 12-hr light-dark cycle at a comfortable temperature.

#### 4.2.2 Electrophysiology.

In the present study, whole-cell patch clamp recordings were performed to record mEPSCs from Layer2/3 pyramidal cells in the mouse PFC. (see supplementary information).

#### 4.2.3 Golgi staining.

FD Rapid GolgiStain Kit was employed to examine the neuromorphopathological alterations. The mouse brain tissue was placed in a mixture of equal volumes of solution A and solution B for two weeks in the dark at room temperature. Tissue was placed in solution C for 4 days. Brain tissue were cut into sagittal slices (100 μm thick) and mounted on slides. Subsequent staining steps are performed according to the instructions. Dendritic spines on the basal dendrites of pyramidal neurons in layer 2/3 of the PFC were selected for statistics. Dendritic spine density was analyzed by Fiji software.

#### 4.2.4 Western-blotting.

As previously described [53], the brain tissues from the prefrontal cortex and hippocampus of mice were collected, and protein was cleaved, extracted, and then the samples were prepared by 6 × loading buffer for the next steps. The specific conditions for separating the proteins, transferred to PVDF membrane, blocking with milk and antibody incubation are described in the Supplementary material. Protein bands were visualized by chemiluminescence and quantified using Fiji software.

#### 4.2.5 Immunofluorescence staining.

After anesthesia, the mice were rapidly perfused with 0.9% saline solution and 4% paraformaldehyde, and the brain tissues were collected. Brain tissues were removed and fixed in 4% paraformaldehyde at 4°C for 16 hours, then dehydrated in 30% sucrose solution. Next, brain tissue were cut into sagittal slices at a thickness of 16 μm. The slices were blocked and incubated with primary and secondary antibody. Images were taken by Zeiss LSM 900 with Airyscan 2 confocal microscope.

#### 4.2.6 Gene overexpression.

Simultaneous overexpression of KIF15 and PSD95 in 293T cell lines, corresponding empty plasmids were used as controls. Lipo8000(Cat. C0533, Beyotime) was used as the transfection reagent, and the experimental procedure was carried out according to the instructions(see supplementary information).

#### 4.2.7 Co-immunoprecipitation.

Protein cleavage and extraction were performed as previously described. The primary antibody was added to the protein lysate and incubated overnight at 4 ° C by shaker, agarose beads were added on the second day and shaken at 4° C overnight, and on the third day the protein was separated from the beads by 95 ° C water bath, the supernatant protein for subsequent Western Blot. Exogenous co-IP directly added protein lysates to magnetic beads with tagged antibodies.

#### 4.2.8 Behavioral assays.

Tail suspension test and Forced swimming test were performed to detect depressive behavior. The manic behavior of mice was detected by open field test. Immobility time and movement trajectory of mice was recorded by EthoVision XT software system.

#### 4.2.9 Microinjection and zebrafish behavior.

Embryonic injections of cas9 protein, *Kif15* sgRNA, and Rat *Kif15* mRNA were performed at the one-cell stage, and the spontaneous swimming behavior of zebrafish larvae was assessed on day 4 post-fertilization. Detailed procedural steps are provided in the Supplementary Materials and Methods.

#### 4.2.10 Statistical analysis.

Data were analyzed by unpaired two tailed Student’s t test, Multiple t tests and expressed as mean±SD. p < 0.05 was considered statistically significant (see supplementary information).

## Supporting information

S1 Text Materials and methods. (DOCX)

S1 Fig Manhattan plot for genome-wide association study (GWAS) in a European population with depression. A: The x-axis represents the chromosomal position, and the y-axis represents the significance on a –log10 scale. The rs3804583 marked in the figure is a variant SNP located in the exon of KIF15 in the population wit depression, rs3804580 and rs7622843 are two SNPs located in the intron variation of the KIF15 gene. The red line represents the genome-wide significance threshold of 5 × 10^−8^ and the blue line 10^−5^. B: The representative co-IP image of PSD95-Flag and KIF15-GFP or KIF15 mutant (C3636A or C3636G) plasmids co-expressed in 293T cells. C: Point mutations in the tail region of KIF15 significantly decreased its binding efficiency to PSD95, n = 3 (three independent experiments). All the data are presented as mean±SD, *P < 0.05, **P < 0.01, two-tailed student’s t test between two groups.
(TIF)

S2 Fig Gene knockout strategy and genotype identification in KO mice. A: *Kif15* gene knockdown strategy diagram; B: Results of mouse genotype identification, M, 2000 bp marker, lane 1、3、5: *Kif15*^*+/-*^ mouse; lane 2: *Kif15*^*-/-*^ mouse; lane 4: *Kif15*^*+/+*^ mouse; C: The knockout of *Kif15* protein was confirmed in the PFC and hippocampus of *Kif15*^*-/-*^ mice in P1.
(TIF)

S3 Fig The distribution of PSD95 and KIF15 in the cortex and hippocampus of 3-week, 6-week, and 11-week *Kif15*^*+/+*^ and *Kif15*^*-/-*^ mice was examined. (TIF)

S4 Fig Emotional changes in *Kif15*^*-/-*^ mice from adolescence to adulthood. A: From 14W to 9M, the time or frequency of entering the open arm of *Kif15*^*-/-*^ mice were significantly lower than those of *Kif15*^*+/+*^ mice. B: At 24 weeks of adulthood, the total distance traveled by *Kif15*^*-/-*^ mice in the open field test was similar to that of *Kif15*^*+/+*^ mice. n = 7 ~ 9 mice in each group, All the data are presented as mean±SD, *P < 0.05, **P < 0.01,two-tailed student’s t test between two groups.
(TIF)

S5 Fig *Kif15* deletion results in abnormal distribution of PSD95 in neuropil. A: Representative images of PSD95 and synaptophysin immunofluorescence staining in DIV18 neurons treated with KIF15 inhibitor (KIF15-IN-1), B: The KIF15-IN-1 significantly reduced the synaptic density of DIV18 neurons, n = 15 neurons per condition. C and D: The distribution of PSD95 in the dendrites of neurons in the PFC region of *Kif15*^*+/+*^ and *Kif15*^*-/-*^ mice. The solid triangular arrows indicate PSD95 particles retained in the dendrites, and the hollow triangular arrows indicate PSD95 particles distributed in the postsynaptic membranes on both sides of the dendrites, n = 3 ~ 4 mice in each group. E: Representative images of immunofluorescence staining showing colocalization of PSD95 and KIF15 or IgG in the postsynaptic region of DIV18 neurons, The solid white arrowheads represent KIF15 fluorescent particles localized at the postsynaptic site. F: The Pearson coefficients for the postsynaptic localization of KIF15 and PSD95 were significantly higher than those of the IgG group, n = 10 neurons per condition. All the data are presented as mean±SD, *P < 0.05, **P < 0.01, ***P < 0.001 two-tailed student’s t test between two groups.
(TIF)

S6 Fig Agarose gel electrophoresis validation of *Kif15*^*-/-*^zebrafish complement; M, 2000 bp marker. (TIF)

S7 Fig The knockdown efficiency of KIF15 in 293T cells was assessed. A: qRT-PCR showed the knockdown efficiency of KIF15 mRNA. B-C: Western Blot showed the knockdown efficiency of KIF15 protein, n =  3 (hree independent experiments), All the data are presented as mean±SD, *P < 0.05, ***P < 0.001 two-tailed student’s t test between two groups.
(TIF)
