# Supplementary material for: Deficiency of Kif15 impairing synaptic development leads to mood disorder in mice
Source: PLoS Genet. 2025 Sep 2;21(9):e1011839. doi: 10.1371/journal.pgen.1011839 (PMC12413082; doi:10.1371/journal.pgen.1011839)
Supplement: S1 Text — (DOCX) [file pgen.1011839.s001.docx]

Materials and Methods

Animals and genotype analysis

*Kif15^-/-^* mice were *Kif15* systemic knockout mice made by CRISPR/Cas9 technology using C57BL/6 as background mice, constructed by GemPharmatech and bred in Laboratory Animal Center of Nantong University. All animals were housed in a constant environment where was maintain on a 12-hr light-dark cycle at a comfortable temperature (20 ~26°C), humidity between 40 and 70%, and with ad libitum access to water and food. In our breeding colony, two heterozygous females and one heterozygous male were mated to obtain homozygotes. The offspring were tail-clipped at P21-P28 and genotyped by PCR using specific forward and reverse primers. The primers for genotyping the *Kif15^-/-^* are as follow: forward: 5- aggattctggaggcagacaggt-3, reverse: 5-gggatggagttcatttagcagag-3; The primers for genotyping the *Kif15^+/+^* are as follow:forward:5-aatataatgggctgagatagagt-3, reverse: 5-tatggaccctacacaaattgc-3. The PCR products for the *Kif15^-/-^* and *Kif15^+/+^* alleles were 634 bp and 219 bp, respectively. All mice experiments were conducted in accordance with the requirements of National Institutes of Health’s Guide for the Care and Use of Laboratory Animals and were approved by Nantong University, Jiangsu.

Primary Neuron Culture

E18 C57BL/6 wildtype and *Kif15^-/-^* pregnant mouse embryos were carefully dissected to isolate the cortex under a stereomicroscope. After trypsin digestion, the cells were suspended in Neurobasal Media (Cat. 21103049, Gibco) complete medium containing 2%B27 (Cat.05711, STEMCELL, Vancouver, Canada), 1% penicillin and 1% streptomycin and 2 mM glutamine prepare a cell suspension. Neurons were plated onto 14 mm round coverslips pre-coated with 0.1 mg/mL poly-L-lysine(Cat.P4832, Sigma) at a density of 30,000 cells per well. The medium was fully replaced every 3–4 days. Neurons cultured with KIF15 inhibitors were routinely maintained in neurobasal complete medium supplemented with 0.2μM KIF15-IN-1(MCE, HY-15948).

In vitro brain slice electrophysiology

Brain slices preparation After mice were anesthetized with isoflurane, mice were then decapitated and brains were quickly removed and chilled in ice-cold hypertonic artificial cerebrospinal fluid (ACSF) containing (in mM) containing 234 sucrose, 3.6 KCl, 1.2 NaH_2_PO_4_∙2H_2_O, 12 Glucose, 25 NaHCO_3_, 1.2 MgCl_2_∙6H_2_O, 2.5 CaCl_2_ and frozen for 1 minute. Brain tissue was cut into coronal sections (300 μm in thickness) in ice-cold hypertonic ACSF using a Vibroslice. Brain slices were subsequently incubated in conventional ACSF containing (in mM) 125 NaCl, 3 KCl, 1.25 NaH_2_PO_4_∙2H_2_O, 10 Glucose, 26 NaHCO_3_, 5 HEPES, 1.2 MgCl_2_∙6H2O, 2.4 CaCl2 for 30 min at 34°C, and then left at room temperature (22 to 25°C) for 1.5h for recovery. All solutions were saturated with 95% O_2_, 5% CO_2_(vol/vol).

Whole-cell patch-clamp recording Neurons were visualized using an orthogonal fluorescence microscope(Olympus, BX51WI) equipped with a 40× water-immersion objective and a sCMOS camera(optiMOS, QImaging). The signals were collected using a MultiClamp 700B amplifier. Whole-cell patch clamp recordings were performed using patch pipettes drawn by a microelectrode puller (P-97) (input resistance: 3–8MΩ). A DigiData 1400A Data Acquisition System was utilized with a low-pass filter at 2 kHz and digitized at 10 kHz, the data was analyzed using Clampfit 10.4 software.

Synaptic transmission Miniature excitability postsynaptic currents (mEPSCs) were recorded in the presence of tetrodotoxin (TTX, 1 μm) and picrotoxin (PTX, 100 μm) with a clamp voltage of -70 mV. The intracellular solution contained (in mM): 121 K-glucose, 20 KCl, 0.2 EGTA, 4 Na_2_ATP, 0.4 GTP-Tris, 2 MgCl_2_, 10 HEPES. TTX was purchased from must (Chengdu,China) bio-technology CO., LTD. PTX was purchased from MedChemExpress company.

Golgi staining

FD Rapid GolgiStain Kit (FD NeuroTechnologies, Inc., USA) was employed to examine the neuromorphopathological alterations. Golgi staining was performed according to the instructions. After mice were anesthetized with isoflurane, mice were then decapitated and brains were quickly removed, the brain tissue was rinsed of blood on the surface of the brain in dd-H_2_O, and placed in a mixture of equal volumes of solution A and solution B (solution A and B are prepared and mixed 24h in advance). Replace with new AB mix overnight and infiltrate for two weeks in the dark at room temperature. Tissue was placed in solution C and replaced with fresh solution C the next day and infiltrated for 4 days. Brain tissue were cut into sagittal slices (100 μm thick) and mounted on slides. Subsequent staining steps are performed according to the instructions. Dendritic spines on the basal dendrites of pyramidal neurons in layer 2/3 of the PFC were selected for statistics. Dendritic spines were manually classified and counted according to the following principles: mushroom(mushroom shaped head with thin neck), filopodia (long, thin with no head), stubby (short with no neck), Cup shaped (with bifurcated head). Dendritic spine density was analyzed by Fiji software.

Western blotting

After mice were anesthetized with isoflurane, the brain was rapidly decapitated and isolated from the bilateral prefrontal cortex and hippocampus. Homogenize in protein lysis buffer containing protease inhibitors and phosphatase inhibitors, and lysed on ice for 30 min. The homogenate was centrifuged at 12,000 rpm for 15 min at 4°C and the supernatant was extracted, and total protein was quantified using a BCA kit. The supernatant was mixed with 6× loading buffer, and after separating the proteins at 7.5% or 10% SDS-PAGE, the proteins were transferred to a 0.2μm PVDF membrane. After blocking with 5% skimmed milk，the PVDF membrane was incubated with primary antibody at 4°C overnight，including anti-PSD95(1:1000，3450, CST)，anti-GluA1(1:1000, 13185S)，anti-NMDAR2A（1:1000，ab169873，abcam），anti-gephyrin（1:500，Cat NO.12681-1-AP, proteintech），β-actin(1:1000，60008-1-Ig，proteintech), anti-GABRB1(1:500, Cat No. 28425-1-AP, proteintech), anti-GABRA1(1:1000, Cat No.12410-1-AP, proteintech). Then they were incubated with horseradish peroxidase-coupled secondary anti-rabbit or anti-mouse antibodies for 2h at room temperature, respectively. Protein bands were visualized by chemiluminescence and quantified using Fiji software.

Immunofluorescence staining

After mice were anesthetized by intraperitoneal injection of compound anesthetic containing pentobarbital, they were intracardially perfused with 0.9% saline and 4% paraformaldehyde, respectively. Brain tissues were removed and fixed in 4% paraformaldehyde at 4°C for 16 hours, then dehydrated in 30% sucrose solution. Brain tissue were cut into sagittal slices using a Lecia sliding-freezing microtome at a thickness of 16 μm. The slices were blocked with 10% normal goat serum for 2 hours at room temperature and then incubated overnight at 4°C with the following primary antibodies: anti-PSD95(1:200,20665-1-AP,proteintech)，anti-MAP2(1:250,67015-1-Ig,proteintech), anti-KIF15(1:400, synthesized by abmart Inc)washed 3× in phosphate-buffered saline (PBS) and then incubated with secondary antibodies for 2 hours at room temperature(Cy3-conjugated goat anti-mouse IgG; 488-conjugated goat anti-rabbit IgG, 647-conjugated goat anti-chicken IgY ; 1:400, Jackson ImmunoResearch). All sections were counterstained with DAPI for 15 minutes (1:2500, Cat. D9542, Sigma). Images were taken by Zeiss LSM 900 with Airyscan 2 confocal microscope. PSD95 fluorescence density in neuronal dendrites was obtained from 3 to 4 mice per group. PSD95 fluorescence density on dendrites was counted using ImageJ in three randomly selected fields of 63 microscope per mouse, and the mean value was taken as an n value[1].

Immunocytochemistry

293T cells were washed three times with warm PBS, fixed with 4% PFA for 15 minutes at room temperature, washed three times with PBS at room temperature, and blocked in blocking solution (Beyotime, P0102) for 1 hour. Primary antibodies (anti-GFP antibody, 1:2000, Abcam, ab13970; anti-Flag antibody, 1:1000, CST, 8146T; anti-MAP2, 1:3000, Biolegend, 822501; anti-PSD95, 1:500, Affinity, #BF8419; anti-synaptophyin, 1；1000， proteintech, Cat NO.17785-1-AP) were incubated at 4°C overnight. After rewarming to room temperature for 10 minutes, the cells were washed three times with PBS, followed by incubation with the corresponding fluorescent secondary antibodies (Cy3-conjugated goat anti-mouse IgG, 488-conjugated goat anti-chicken IgY, Jackson ImmunoResearch Laboratories, 1:800) for 1 hour and 45 minutes at room temperature. DAPI (Cat. D9542, Sigma, 1:2500) was applied for 15 minutes, and the slides were imaged using confocal microscopy after sealing. The fluorescence intensity of PSD95 on the cell membrane was quantified using ImageJ software.

Gene overexpression

Simultaneous overexpression of KIF15 and PSD95 in 293T cell lines, corresponding empty plasmids were used as controls. GFP-KIF15, Flag-PSD95 or mCherry-PSD95 and the corresponding empty plasmids were designed and synthesized by General Biol, Inc. Lipo8000(Cat. C0533, Beyotime) was used as the transfection reagent, and the experimental procedure was carried out according to the instructions. At 48 h of transfection, ice-cooled protein lysate buffer was used to lysed the cells, and the protein concentration was determined by BCA kit, and the subsequent steps of Western-blotting experiments were performed as before. The primary antibodies were incubated as follows: anti-GFP (1:5000, ab13970,abcam), anti-flag(1:1000,R24091,zenbio).

Small interfering RNA (siRNA) transfection

KIF15 small interfering RNA was transfected into 293T cells, while the corresponding negative control siRNA was transfected into the control group. The siRNAs were designed and synthesized by GenePharma (Suzhou, China), and the sequences of the negative control siRNA and Kif15 siRNA (siKIF15) are listed in S1 Table . Lipo8000(Cat. C0533, Beyotime) was used as the transfection reagent, and the procedure was performed according to the instructions. mRNA expression levels were measured by qRT-PCR 24 hours post-transfection, while protein expression were assessed by Western Blot 48 hours post-transfection.

Quantitative real-time polymerase chain reaction (qRT-PCR)

Approximately 1μg of total RNA was extracted from cells using the Super FastPure Cell RNA Isolation kit（RC102-01, Vazyme, Nanjing, China）, reverse transcribed into cDNA using the HiScript II 1st Strand cDNA Synthesis kit (Cat. R312-02, Vazyme), and real-time quantitative PCR was performed with a SYBR Green qPCR kit (Cat. Q712-02, Vazyme). The primers were designed and synthesized by GenScript (Nanjing, China). Relative mRNA normalized by GAPDH RNA was calculated using a 2^− ΔΔCt^ method The primer sequences are provided in S1 Table.

Live cell imaging for FRAP

293T cells were co-transfected with Kif15-GFP and PSD95-mCherry plasmids for 30 hours and stained with DAPI (1:2500, Cat. D9542, Sigma) for 18 hours. The Tubulin-Tracker Deep Red Staining Kit for Living Cells (C2215S) was used to stain the cells 48 hours post-transfection for 40 minutes at 37°C. The staining solution was then discarded, and the cells were washed three times with dilution containing 1% staining enhancer. High-glucose DMEM complete medium (containing 10% FBS, 1% penicillin-streptomycin, and amphotericin) was added, and live cell imaging was performed using confocal microscopy. GFP and mCherry signals were photobleached with 100% 488 nm and 561 nm lasers, respectively. After photobleaching, 20 additional images were captured using the same imaging parameters.

Co-immunoprecipitation

P1 mice were harvested and brains were quickly removed after decapitation, and the cerebral cortex was dissected on ice. Tissues were placed in IP lysate(Pierce IP lysis，Thermo Fisher，87787) containing protease inhibitors and phosphatase inhibitors, homogenized and lysed on ice for 30 minutes, centrifuge the supernatant at 12000rpm for 15min at 4°C. After the protein concentration was determined by the BCA kit, the corresponding amount of primary antibody was added to the protein lysate overnight at 4°C on a shaker according to the recommended ratio in the antibody instruction manual, and IgG was used as a negative control. The next day, the appropriate amount of protein A agarose beads (20333, Thermo scientific) was added and shaken overnight at 4°C. On the third day, the protein-antibody-bead complex was collected after centrifugation at 4°C, 2×loading buffer was added, and the beads and protein were separated at 95°C. After centrifugation, the supernatant was taken and the beads were discarded, and the subsequent Western-blotting assay was performed, and the experimental steps were as before. Exogenous co-IP directly added protein lysates to magnetic beads with tagged antibodies (anti-GFP magnetic beads, P2132; anti-Flag magnetic beads, P2115, beyotime). After shaking overnight at 4°C, protein bead complexes were collected, 5×loading buffer was added, and subsequent steps were performed as before.

Behavioral Assays

Tail suspension test The tail of the animal was suspended from a bar about 40cm above the ground for 6min. The absence of any active upward behavior of the mouse body was regarded as immobility. Immobility time at the last 4 minutes of mice was recorded using the EthoVision XT software system.

Forced swimming test Mice were individually placed in a transparent cylinder (12cm diameter x 30cm height) in 20cm water depth for 6 min swimming at 22±1 ° C. Mouse immobility time is the time that the body of the mouse floats on the water without struggle, and only slight limb movements appear to keep the head afloat. Water was changed between subjects. Immobility time at the last 4 minutes of mice was recorded using the EthoVision XT software system.

Open field test An open field box with a base area of 50cm×50cm and a height of 40cm was used in the open field experiment, and the central area was defined as 16.7×16.7cm square. The mice were placed in the open field box from the middle area, and the total distance and the time of crossing the center area were recorded for 10 minutes. The animal movement trajectory was recorded by EthoVision XT software system.

Microinjection of Zebrafish Embryos

At the one-cell stage, a mixture containing 300 ng/μl Cas9 protein(Z03388, GenScript Biotech Corporation) and 100 ng/μl kif15 sgRNA was injected into zebrafish embryos, with 1 nl of the solution per embryo, to construct the zebrafish kif15 full knockout model. The control group was injected with the same amount of Cas9 protein only. The single overexpression group received only Rat kif15 mRNA at a concentration of 150 ng/μl, while the knockdown and overexpression groups were injected simultaneously with the same amount of Cas9 protein, kif15 sgRNA, and Rat Kif15 mRNA. Borosilicate glass capillaries (Sutter, USA) were used for the injection, which were pulled using a P-97 micropipette puller (Sutter, USA) and connected to an IM-400 pneumatic microsyringe (Narishige, Japan) for the injection process. Rat Kif15 mRNA was transcribed and synthesized using the mMessage mMachine Kit (Thermo Fisher Scientific). Rat Kif15 mRNA[2] and Kif15 sgRNA[3] sequences were referenced from our previous studies.

Zebrafish Behavior

On days 4 post-fertilization, zebrafish larvae were imaged using the Noldus DanioVision system, and behavioral tests were performed using the animal movement tracking system (https://www.noldus.com/). The larvae were placed in 24-well plates, with each juvenile occupying one well. Spontaneous swimming behavior was observed for 30 minutes.

Intrauterine injection of adenovirus overexpressing Kif15(Adv-Kif15):

*Kif15^-/-^* mice at E15 were anesthetized using isoflurane gas and placed in the supine position. After removing the abdominal villi, the abdomen was sterilized with iodophor, and the abdominal skin and uterine wall were carefully incised with sterilized small surgical scissors. The fetal mice were transferred from the uterine cavity to gauze, and their heads were fixed with forceps. Adenovirus overexpressing Kif15(constructed and synthesized by Shanghai Genechem Co.,Ltd) was injected into the cortex of the fetal mice at a volume of 4 μl (virus titer: 10^9 PFU/ml). After completing the injections, the uterine and abdominal walls were sutured sequentially, and the wounds were closed with wound clips. The mice were harvested at postnatal day 1 for staining.

Statistical Analysis

OriginPro 2018 software (OriginLab Corporation, USA), Rstudio and GraphPad Prism 9 (GraphPad Software, Inc., USA) were used for the statistical analyses and graphing. The two-tailed unpaired t test was used to compare the two groups in accordance with normal distribution, otherwise the nonparametric test was used, Multiple t test in different morphology spine group. Data following a normal distribution are presented as mean±SD. Statistically significant difference was indicated as follows: **P < 0.01, and *P < 0.05 and not significant (ns.).

[1] Shao CY, Mirra SS, Sait HB, Sacktor TC, Sigurdsson EM. Postsynaptic degeneration as revealed by PSD-95 reduction occurs after advanced Abeta and tau pathology in transgenic mouse models of Alzheimer's disease. Acta Neuropathol. 2011;122(3):285-92.

[2] Feng J, Hu Z, Chen H, Hua J, Wu R, Dong Z, et al. Depletion of kinesin-12, a myosin-IIB-interacting protein, promotes migration of cortical astrocytes. J Cell Sci. 2016;129(12):2438-47.

[3] Dong Z, Wu S, Zhu C, Wang X, Li Y, Chen X, et al. Clustered Regularly Interspaced Short Palindromic Repeats (CRISPR)/Cas9-mediated kif15 mutations accelerate axonal outgrowth during neuronal development and regeneration in zebrafish. Traffic. 2019;20(1):71-81.

**Supplementary tables**

S1 Table. Nucleotide sequences used in this study.

| Usage | Target | Sequence(5’-3’) |
| --- | --- | --- |
| qRT-PCR | GAPDH primer-F | GAGAAGGCTGGGGCTCATTT |
|  | GAPDH primer-R | AGTGATGGCATGGACTGTGG |
|  | Human-KIF15 primer-F | AAAACTGGCTCCTGCAAGGT |
|  | Human-KIF15 primer-R | CACTTTGGACTTCTTCTAGGTCTG |
|  | 18S primer-F | TCGCTAGTTGGCATCGTTTATG |
|  | 18S primer-R | CGGAGGTTCGAAGACGATCA |
|  | Rat-Kif15 primer-F | CAAATGCAGGAGCTGTTCTC |
|  | Rat-Kif15 primer-R | TCTCTTGACTCTCTTTGAATGTGC |
| siRNA | Control sense | UUCUCCGAACGUGUCACGUTT |
|  | Control antisense | ACGUGACACGUUCGGAGAATT |
|  | KIF15 sense | CCAAGUGAUUACAGCACUUTT |
|  | KIF15 antisense | AAGUGCUGUAAUCACUUGGTT |
|  | KIF15 sense | CCUAUCAACACUUAACUUUTT |
|  | KIF15 antisense | AAAGUUAAGUGUUGAUAGGTT |
