# Supplementary figures and images for: Deficiency of Kif15 impairing synaptic development leads to mood disorder in mice
Source: PLoS Genet. 2025 Sep 2;21(9):e1011839. doi: 10.1371/journal.pgen.1011839 (PMC12413082; doi:10.1371/journal.pgen.1011839)

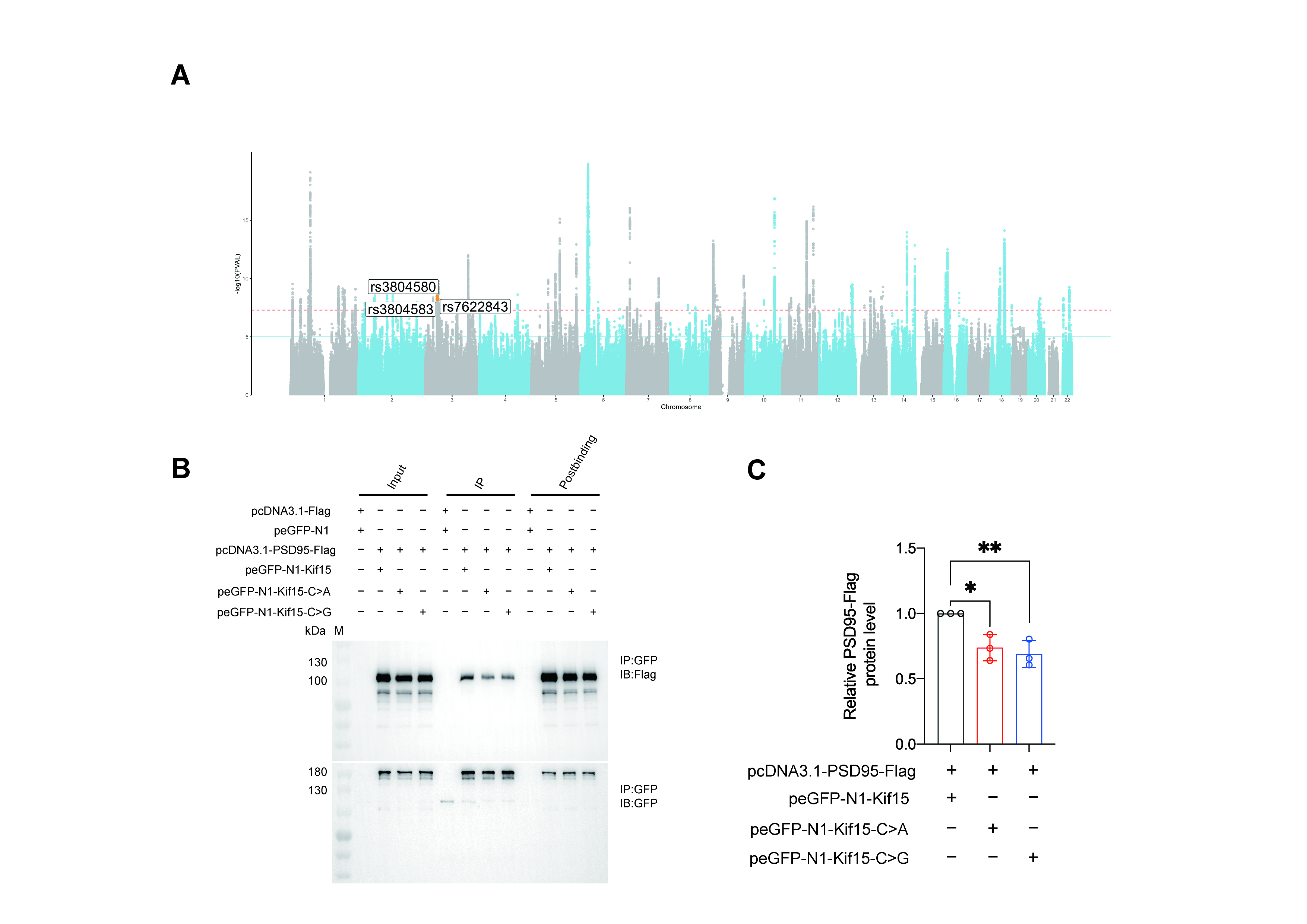

Supplement: S1 Fig — A: The x-axis represents the chromosomal position, and the y-axis represents the significance on a –log10 scale. The rs3804583 marked in the figure is a variant SNP located in the exon of KIF15 in the population wit depression, rs3804580 and rs7622843 are two SNPs located in the intron variation of the KIF15 gene. The red line represents the genome-wide significance threshold of 5 × 10^−8^ and the blue line 10−5. B: The representative co-IP image of PSD95-Flag and KIF15-GFP or KIF15 mutant (C3636A or C3636G) plasmids co-expressed in 293T cells. C: Point mutations in the tail region of KIF15 significantly decreased its binding efficiency to PSD95, n = 3 (three independent experiments). All the data are presented as mean±SD, *P < 0.05, **P < 0.01, two-tailed student’s t test between two groups. (TIF) [file pgen.1011839.s002.tif]

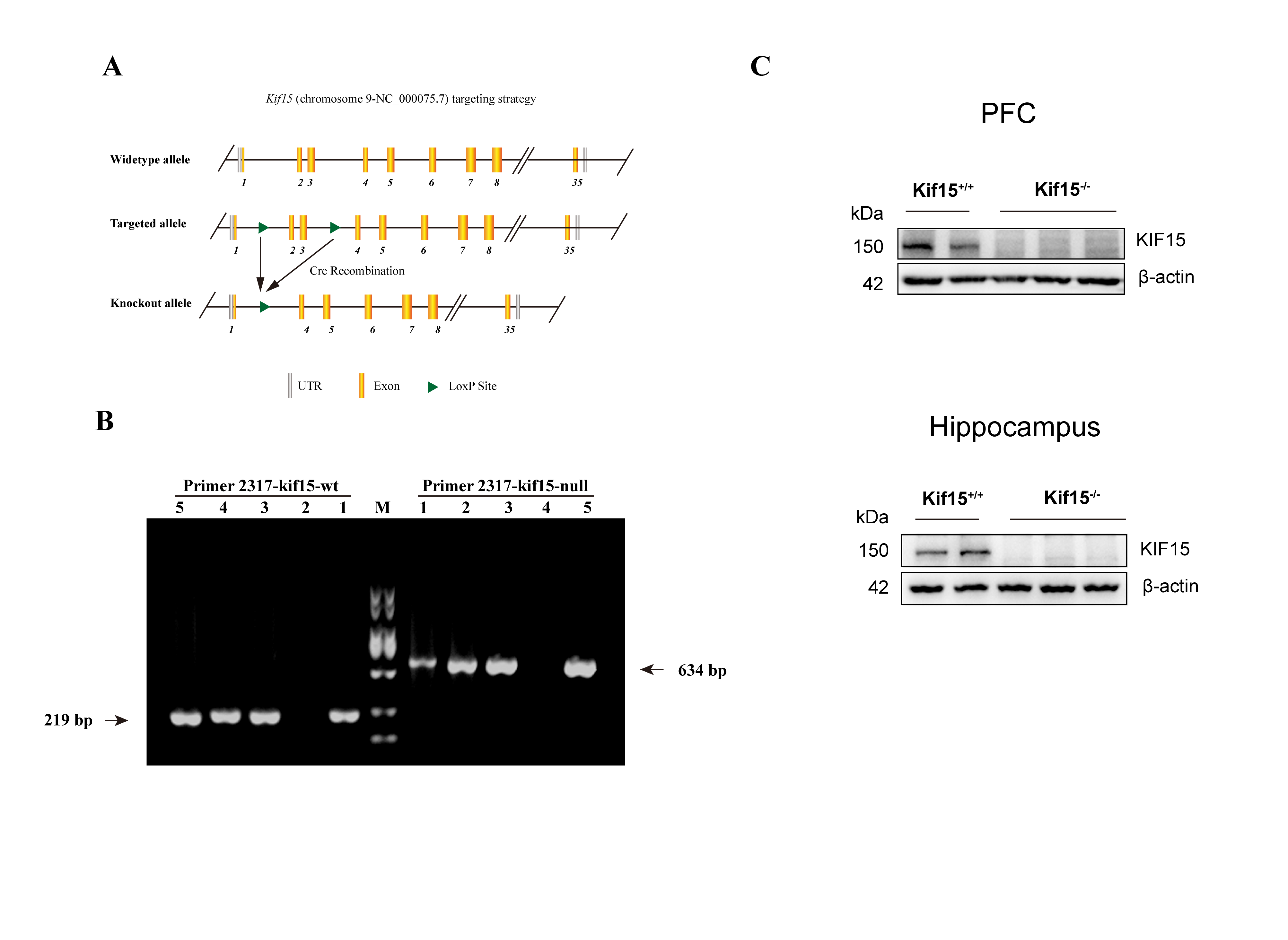

Supplement: S2 Fig — A: Kif15 gene knockdown strategy diagram; B: Results of mouse genotype identification, M, 2000 bp marker, lane 1、3、5: Kif15+/- mouse; lane 2: Kif15-/- mouse; lane 4: Kif15+/+ mouse; C: The knockout of Kif15 protein was confirmed in the PFC and hippocampus of Kif15-/- mice in P1. (TIF) [file pgen.1011839.s003.tif]

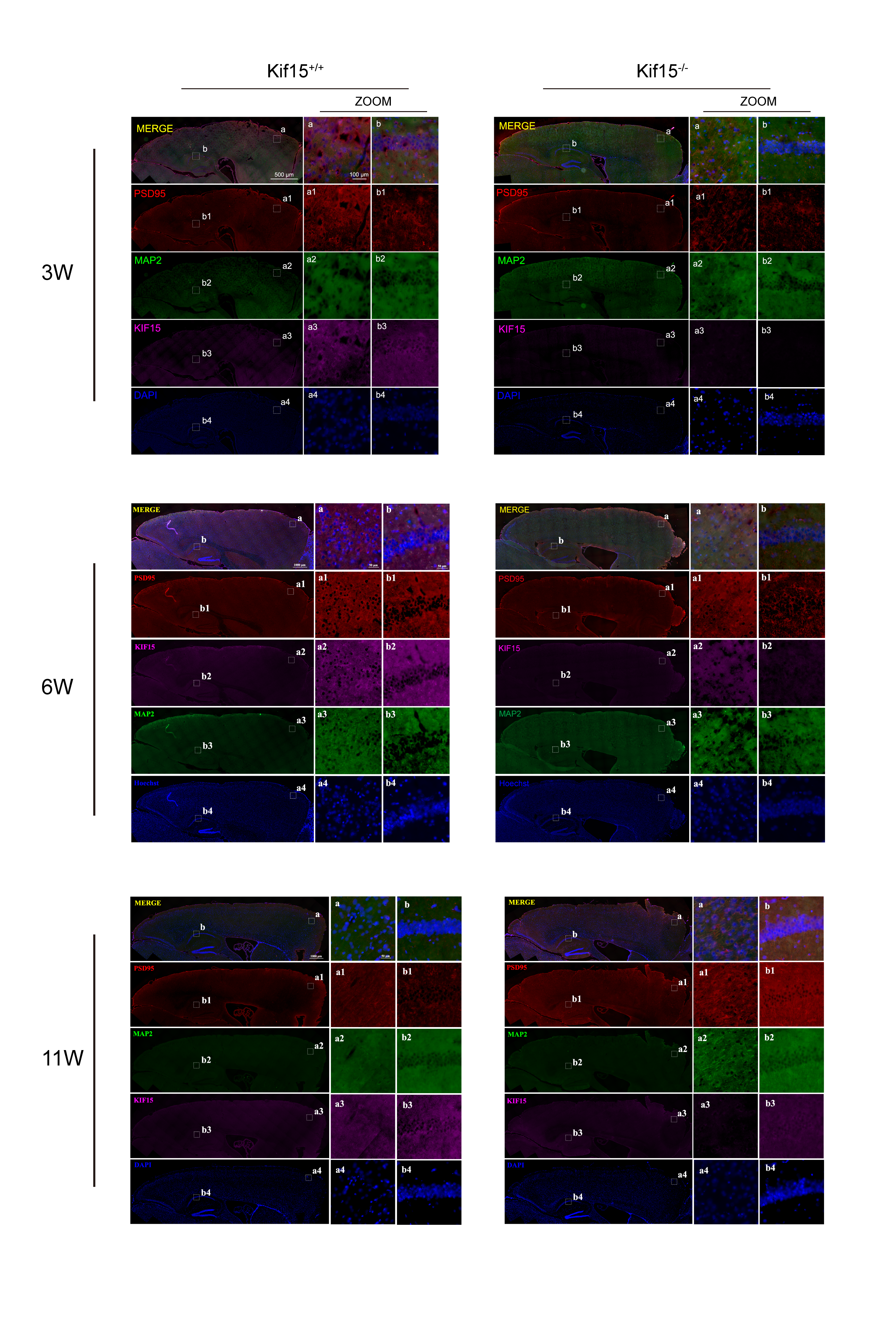

Supplement: S3 Fig — (TIF) [file pgen.1011839.s004.tif]

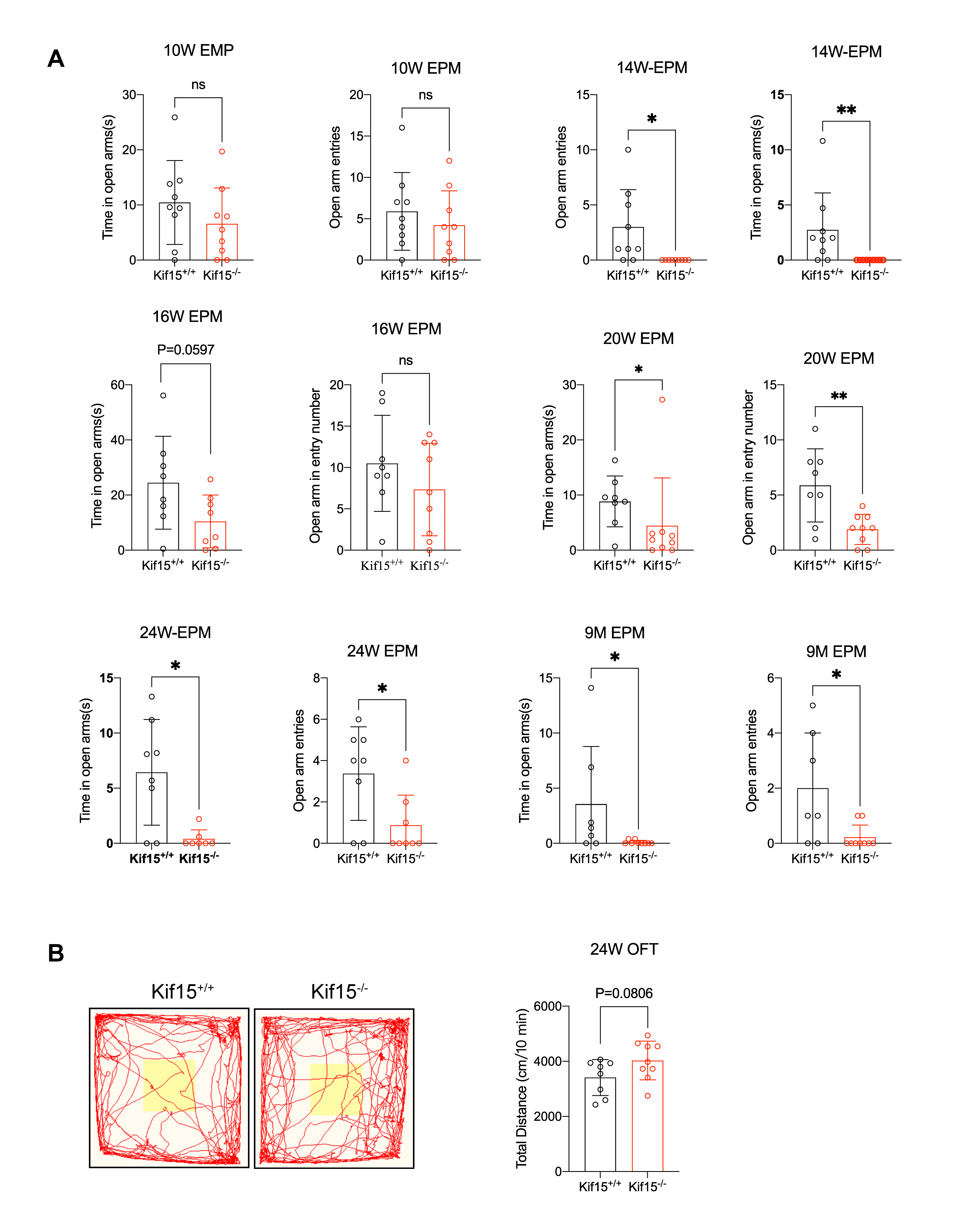

Supplement: S4 Fig — A: From 14W to 9M, the time or frequency of entering the open arm of Kif15-/- mice were significantly lower than those of Kif15+/+ mice. B: At 24 weeks of adulthood, the total distance traveled by Kif15-/- mice in the open field test was similar to that of Kif15+/+ mice. n = 7 ~ 9 mice in each group, All the data are presented as mean±SD, *P < 0.05, **P < 0.01,two-tailed student’s t test between two groups. (TIF) [file pgen.1011839.s005.tif]

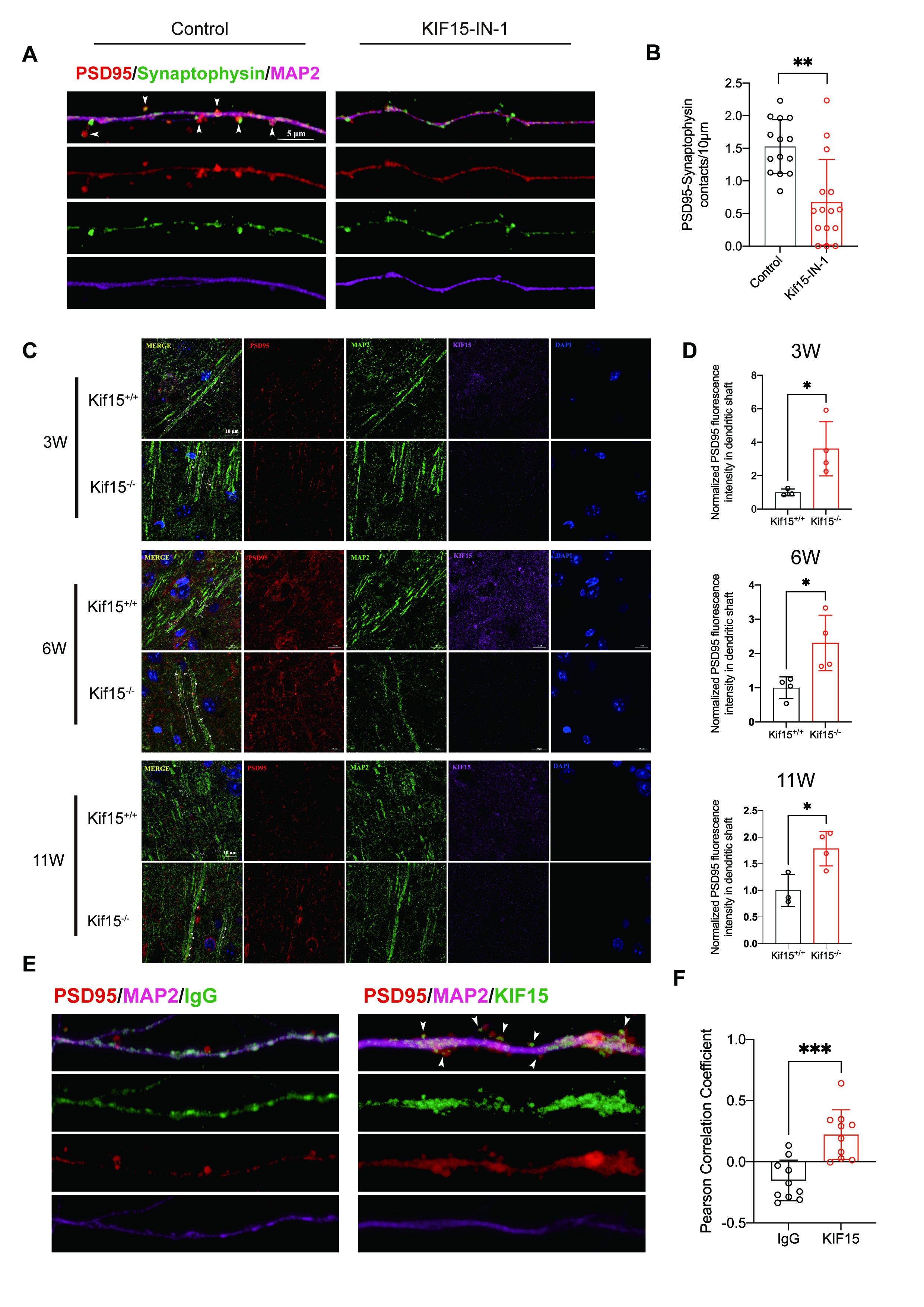

Supplement: S5 Fig — A: Representative images of PSD95 and synaptophysin immunofluorescence staining in DIV18 neurons treated with KIF15 inhibitor (KIF15-IN-1), B: The KIF15-IN-1 significantly reduced the synaptic density of DIV18 neurons, n = 15 neurons per condition. C and D: The distribution of PSD95 in the dendrites of neurons in the PFC region of Kif15+/+ and Kif15-/- mice. The solid triangular arrows indicate PSD95 particles retained in the dendrites, and the hollow triangular arrows indicate PSD95 particles distributed in the postsynaptic membranes on both sides of the dendrites, n = 3 ~ 4 mice in each group. E: Representative images of immunofluorescence staining showing colocalization of PSD95 and KIF15 or IgG in the postsynaptic region of DIV18 neurons, The solid white arrowheads represent KIF15 fluorescent particles localized at the postsynaptic site. F: The Pearson coefficients for the postsynaptic localization of KIF15 and PSD95 were significantly higher than those of the IgG group, n = 10 neurons per condition. All the data are presented as mean±SD, *P < 0.05, **P < 0.01, ***P < 0.001 two-tailed student’s t test between two groups. (TIF) [file pgen.1011839.s006.tif]

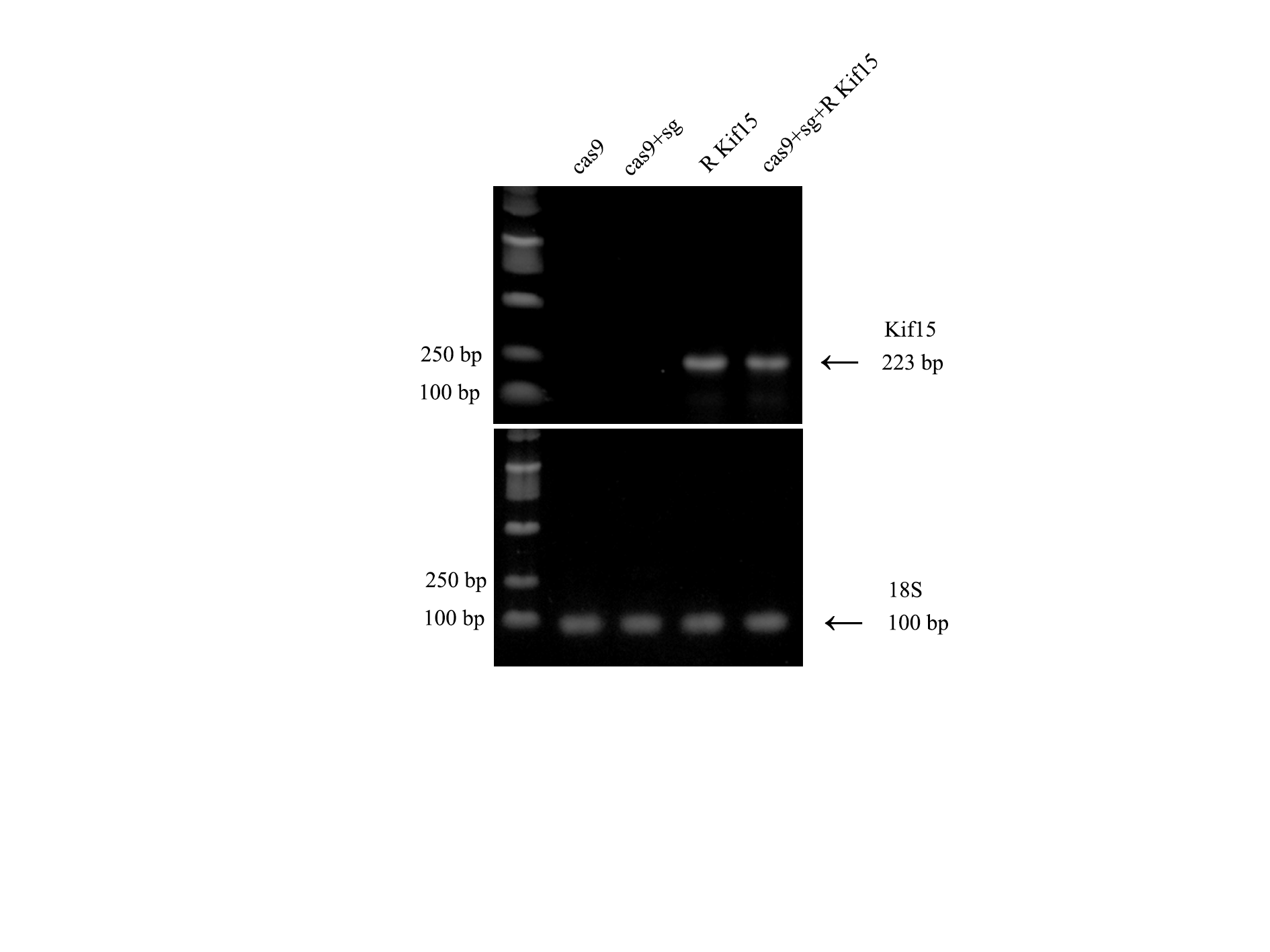

Supplement: S6 Fig — (TIF) [file pgen.1011839.s007.tif]

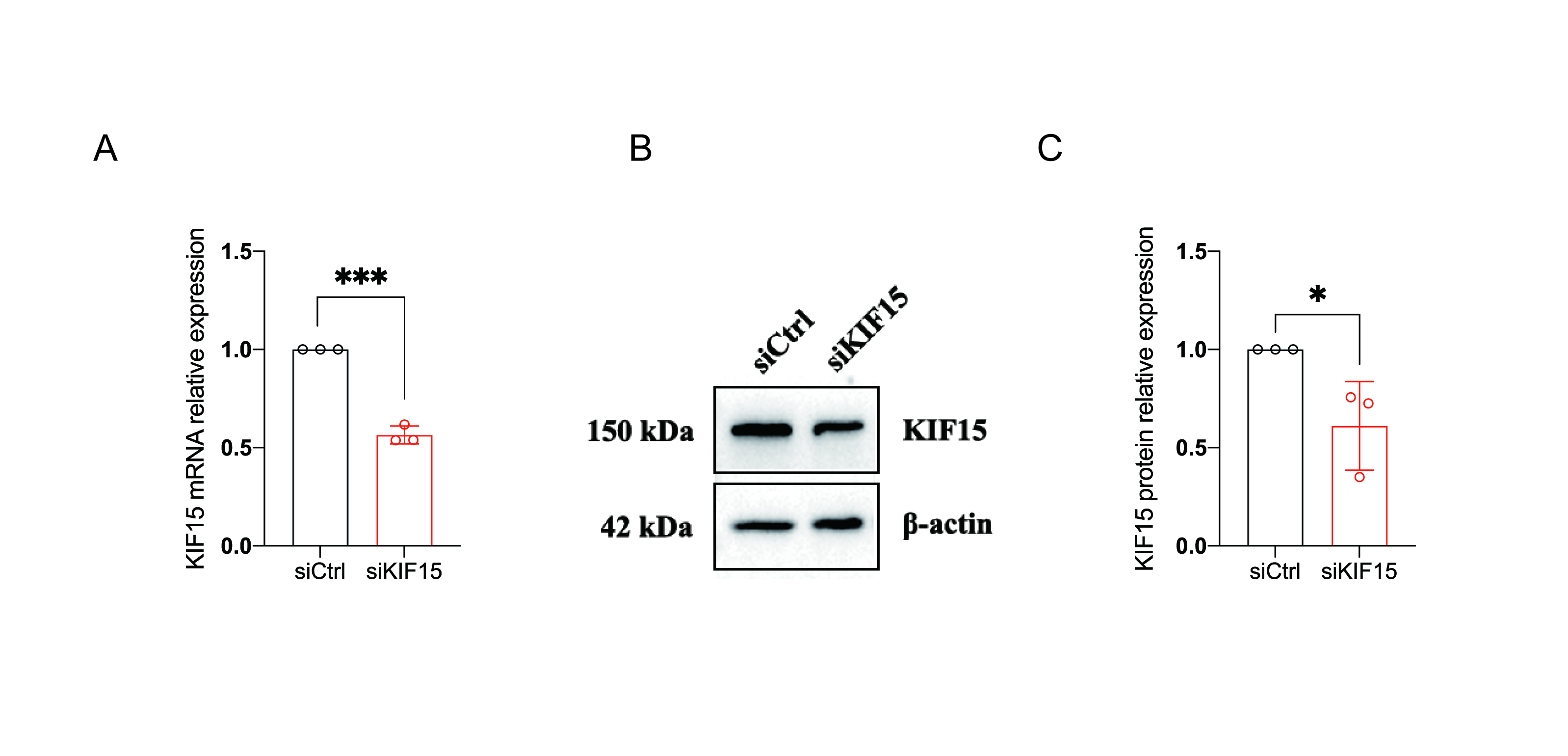

Supplement: S7 Fig — A: qRT-PCR showed the knockdown efficiency of KIF15 mRNA. B-C: Western Blot showed the knockdown efficiency of KIF15 protein, n = 3 (hree independent experiments), All the data are presented as mean±SD, *P < 0.05, ***P < 0.001 two-tailed student’s t test between two groups. (TIF) [file pgen.1011839.s008.tif]
